# An RNF12-USP26 amplification loop drives germ cell specification and is disrupted by disease-associated mutations

**DOI:** 10.1126/scisignal.abm5995

**Published:** 2022-07-12

**Authors:** Anna Segarra-Fas, Carmen Espejo-Serrano, Francisco Bustos, Houjiang Zhou, Feng Wang, Rachel Toth, Thomas Macartney, Ingolf Bach, Gino Nardocci, Greg M. Findlay

**Affiliations:** 1 The MRC Protein Phosphorylation and Ubiquitylation Unit, School of Life Sciences, The University of Dundee, Dundee DD1 5EH, UK; 2 Department of Molecular, Cell and Cancer Biology, University of Massachusetts Chan Medical School, Worcester, MA 01605, USA; 3 School of Medicine, Faculty of Medicine, Universidad de los Andes, Santiago, Chile; 4 Program in Molecular Biology and Bioinformatics, Center for Biomedical Research and Innovation (CIIB), Universidad de los Andes, Santiago, Chile; 5 IMPACT, Center of Interventional Medicine for Precision and Advanced Cellular Therapy, Santiago, Chile

## Abstract

The E3 ubiquitin ligase RNF12 plays essential roles during development, and the gene encoding it, *RLIM*, is mutated in the X-linked human developmental disorder Tonne-Kalscheuer syndrome (TOKAS). Substrates of RNF12 include transcriptional regulators such as the pluripotency-associated transcriptional repressor REX1. Using global quantitative proteomics in male mouse embryonic stem cells (mESCs), we identified the deubiquitylase USP26 as a putative downstream target of RNF12 activity. RNF12 relieved REX1-mediated repression of *Usp26*, leading to an increase in USP26 abundance and the formation of RNF12-USP26 complexes. Interaction with USP26 prevented RNF12 autoubiquitylation and proteasomal degradation, thereby establishing a transcriptional feed-forward loop that amplified RNF12-dependent derepression of REX1 targets. We showed that the RNF12-USP26 axis operated specifically in mouse testes and was required for the expression of gametogenesis genes and for germ cell differentiation in vitro. Furthermore, this RNF12-USP26 axis was disrupted by *RLIM* and *USP26* variants found in TOKAS and infertility patients, respectively. This work reveals synergy within the ubiquitylation cycle that controls a key developmental process in gametogenesis and that is disrupted in human genetic disorders.

## Introduction

Ubiquitylation is a posttranslational modification that serves as a cellular control system by altering the activity, function, subcellular localization, and/or stability of protein targets (1). Depending on linkage topology, ubiquitylation can target proteins for proteasomal degradation, control signaling activity, or promote complex assembly (2). A key role of protein ubiquitylation is the regulation of developmental cell fate decisions (3–5), and as such ubiquitylation components are frequently disrupted in developmental disorders. This is exemplified by the RING E3 ubiquitin ligase RNF12, encoded by the gene *RLIM*, which is mutated in the X-linked developmental disorder Tonne-Kalscheuer syndrome (TOKAS) (6–8). TOKAS affects hemizygous males and is characterized by intellectual disability and associated craniofacial abnormalities (6–8), with syndromic features including urogenital abnormalities and hypogenitalism (6–8). These data suggest key functions for RNF12 in neurological and genital development that are disrupted in TOKAS. Recent reports suggest a further role for RNF12 in fertility, because heterozygous female carriers of RNF12 TOKAS variants exhibit fertility defects (6), whereas RNF12-deficient male mice display impaired spermiogenesis (9).

RNF12 variants associated with TOKAS display impaired catalytic E3 ubiquitin ligase activity leading to defects in substrate ubiquitylation (6, 10). Thus, a major molecular function of RNF12 that is disrupted by TOKAS variants is the regulation of gene expression by ubiquitylation and proteasomal degradation of transcriptional regulators. RNF12 has been shown to ubiquitylate the transcriptional regulators SMAD7 (11), REX1 (12) and CLIM (13), amongst others. As a result, RNF12 plays a key role in coordinating developmental gene expression, with functions including gene dosage compensation by imprinted X-chromosome inactivation (14–16) and transcriptional repression of neurodevelopmental genes (17). However, beyond these notable examples, RNF12 regulation and function have not been systematically investigated at the molecular level, which may shed light on further key developmental functions that are disrupted in disease.

Here, we deployed quantitative proteomics and CRISPR-Cas9 gene editing in embryonic stem cells to systematically elucidate key molecular functions of RNF12. A major feature of RNF12-dependent proteome dynamics was the induction of the X-linked deubiquitylase USP26. Mechanistically, the RNF12-mediated ubiquitylation and resulting degradation of the transcriptional repressor REX1 derepressed *Usp26* expression, which drove a biochemical interaction between USP26 and RNF12. RNF12-USP26 complexing inhibited RNF12 autoubiquitylation, thereby stabilizing RNF12 to establish a feed-forward loop that amplified RNF12-dependent developmental gene expression. This RNF12-USP26 amplification loop specifically operated in testes and promoted efficient expression of genes that were associated with gametogenesis and germ cell differentiation in vitro, including *Dazl* (18), *Usp9y* (19), and *Dppa3* (20). Finally, we showed that the RNF12-USP26 axis was disrupted by *RLIM* variants from TOKAS patients, whereas RNF12 stabilization and downstream transcriptional regulation was disrupted by *USP26* gene variants identified from azoospermia patients. Taken together, our results uncovered molecular synergy within the ubiquitylation cycle which controls germ cell development and is disrupted in diverse human genetic disorders.

## Results

### RNF12 activity drives expression of USP26 by promoting the degradation of REX1

**Fig. 1:**
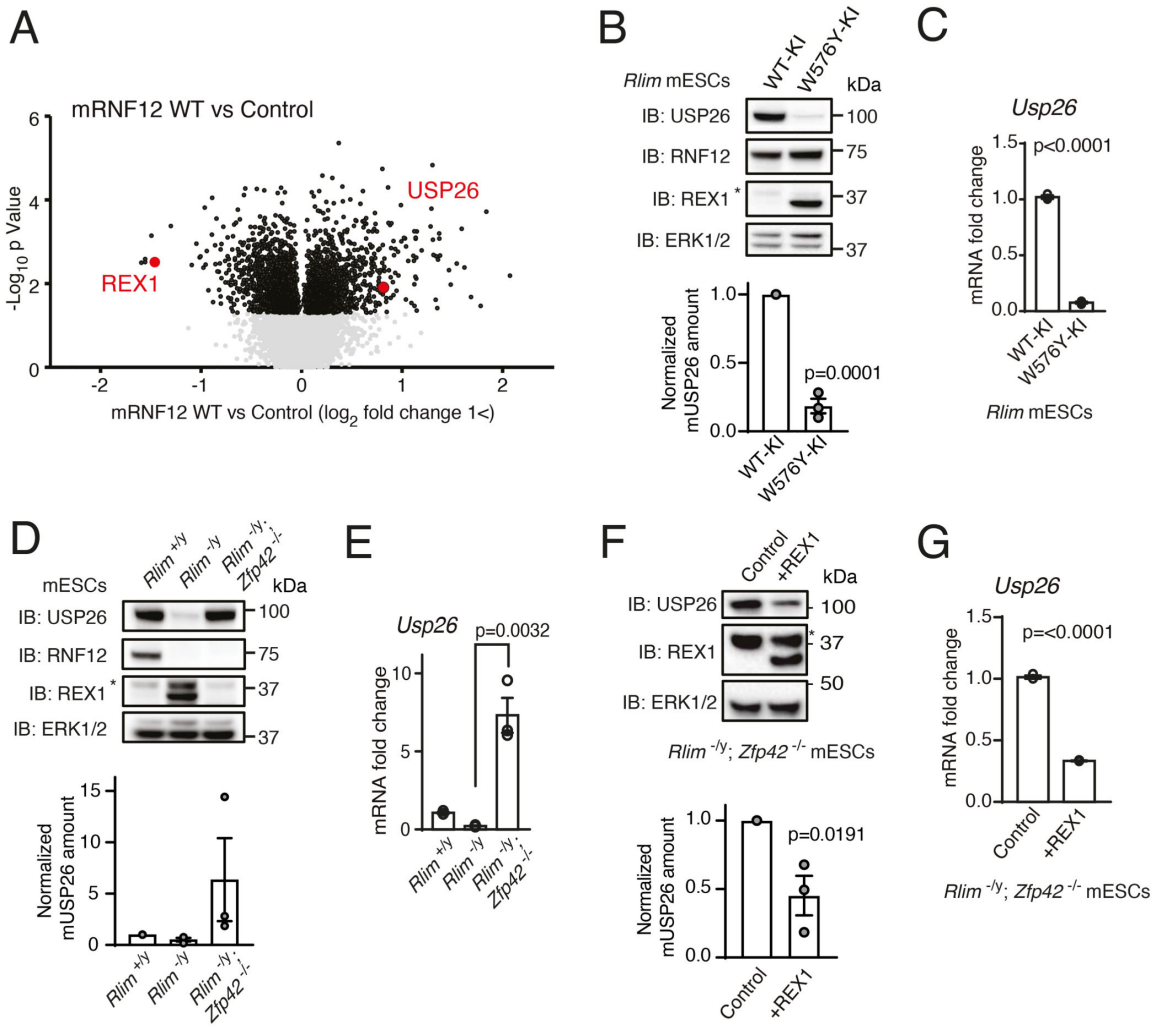
The RNF12-REX1 signaling axis drives transcription of the X-linked gene *Usp26*. **(A**) Quantitative proteomic analysis comparing RNF12-deficient *(Rlim^-/y^)* mESCs expressing empty vector (Control) or wild-type (WT) mouse RNF12 (mRNF12). USP26 and the known RNF12 substrate REX1 are highlighted. (**B**) Immunoblotting (IB) and quantification of RNF12, REX1, and USP26 in control RNF12 WT knock-in (WT-KI) and RNF12 catalytic mutant knock-in (W576Y-KI) mESCs. ERK1/2 is a loading control. The asterisk marks a non-specific band. Data represented as mean ± S.E.M. Statistical significance was determined by paired T-test; confidence level 95%. (**C**) Quantification of *Usp26* mRNA in RNF12 WT-KI and W576Y-KI mESCs by qRT-PCR. Data represented as mean ± S.E.M. Statistical significance was determined by paired T-test; confidence level 95%. (**D**) Immunoblotting and quantification of RNF12, REX1, and USP26 in control (*Rlim*^+/y^), *Rlim^-/y^*, and RNF12;REX1 double knockout *(Rlim^-/y^; Zfp42^-/-^)* mESCs. Data represented as mean ± S.E.M. (**E**) Quantification of *Usp26* mRNA in *Rlim^+/y^, Rlim^-/y^*, and *Rlim^-/y^; Zfp42^-/-^* mESCs by qRT-PCR analysis. Data represented as mean ± S.E.M. Statistical significance was determined by paired T-test; confidence level 95%. (**F**) Immunoblotting and quantification of USP26 and REX1 in *Rlim^-/y^; Zfp42^-/-^* mESCs expressing empty vector (Control) or REX1. Data represented as mean ± S.E.M. Statistical significance was determined by paired T-test; confidence level 95%. (**G**) Quantification of *Usp26* mRNA in *Rlim^-/y^*; *Zfp42^-/-^* mESCs expressing empty vector (Control) or REX1 by qRT-PCR analysis. Data represented as mean ± S.E.M. Statistical significance was determined by paired T-test; confidence level 95%. For all panels, n = 3 independent experiments.

In order to identify previously unappreciated molecular functions of the RNF12 signaling pathway, we performed a comprehensive mapping of RNF12-dependent proteome dynamics in male mouse embryonic stem cells (mESCs) using quantitative proteomics. *Rlim^-/y^* mESCs were reconstituted with either empty vector or wild-type (WT) mouse RNF12 (mRNF12) and relative protein abundances determined using isobaric tag mass spectrometry (Fig. 1A). Proof of principle for this approach was demonstrated by identification of a key RNF12 substrate, the transcriptional repressor REX1 (also known as ZFP42) (Fig. 1A), the abundance of which was suppressed by the expression of WT mRNF12 as predicted. The cohort of proteins that were significantly induced by WT mRNF12 included the X-chromosome-encoded deubiquitylase USP26 (Fig. 1A), which was reported in a previous study of RNF12 proteomics (21) (fig. S1). USP26 and RNF12 exhibit substantial functional overlap, including regulation of SMAD7 ubiquitylation (22) and pluripotency (23). USP26 is implicated in male fertility (24–28), another reported function of RNF12 (9). Thus, we hypothesized that USP26 plays a functional role in the RNF12 signaling network.

To confirm that USP26 protein abundance was increased by endogenous RNF12 E3 ubiquitin ligase activity, we employed control WT RNF12 knock-in mESCs (WT-KI) and RNF12 knock-in mESCs encoding a mutant form with impaired catalytic activity (W576Y-KI) (17). Consistent with disrupted RNF12 E3 ubiquitin ligase activity, RNF12 W576Y-KI mESCs displayed accumulation of the RNF12 substrate REX1 (Fig. 1B). USP26 protein was present in control RNF12 WT-KI mESCs, but abundance was substantially reduced in RNF12 W576Y-KI mESCs (Fig. 1B), supporting the mass spectrometry results. These data confirm that USP26 protein was dynamically increased by RNF12 E3 ubiquitin ligase activity. Because RNF12 ubiquitylates REX1 and other developmental transcriptional regulators, we hypothesized that RNF12 E3 ubiquitin ligase activity increases the expression of *Usp26*. Indeed, *Usp26* mRNA was abundant in RNF12 WT-KI mESCs and reduced in RNF12 W576Y-KI mESCs (Fig. 1C). Therefore, RNF12 E3 ubiquitin ligase activity increased *Usp26* mRNA, most likely by promoting *Usp26* gene transcription.

We next addressed the mechanism by which RNF12 drives *Usp26* expression. The REX1 transcriptional repressor is a major RNF12 substrate in mESCs (Fig. 1A) and plays a critical role in the initiation of imprinted X-chromosome inactivation (XCI) (12, 17, 21, 29). During imprinted XCI, RNF12-dependent ubiquitylation and degradation of REX1 relieves transcriptional repression of the long non-coding RNA *Xist* (12, 21). Thus, we tested whether RNF12 also promotes *Usp26* expression by relieving REX1-mediated transcriptional repression. To this end, we took advantage of a series of CRISPR-Cas9 knockout mESC lines (17) comprising WT *(Rlim^+/y^)*, RNF12 knockout *(Rlim^-/y^)*, and RNF12;REX1 double knockout *(Rlim-/y Zfp42^-/-^)* (Fig. 1D). As expected, RNF12 knockout led to increased REX1 protein in these cells, which was reversed by REX1 disruption (Fig. 1D). USP26 protein was reduced by RNF12 knockout and restored in RNF12;REX1 double knockout mESCs (Fig. 1D), suggesting that REX1 is the key RNF12 substrate in the regulation of USP26. *Usp26* mRNA followed a similar pattern (Fig. 1E), suggesting that REX1 may directly inhibit *Usp26* gene transcription. In that regard, RNF12;REX1 double knockout mESCs produced more *Usp26* mRNA than did WT mESCs (Fig. 1E), consistent with REX1-mediated transcriptional repression of *Usp26*. Restoration of REX1 in RNF12;REX1 double knockout mESCs reduced USP26 protein (Fig. 1F) and *Usp26* mRNA (Fig. 1G), providing evidence that REX1 is epistatic to RNF12 for *Usp26* transcription. Taken together, these results confirm that degradation of the REX1 transcriptional repressor is a primary mechanism by which RNF12 drives *Usp26* gene expression.

### RNF12 and USP26 form a complex in mESCs

**Fig. 2:**
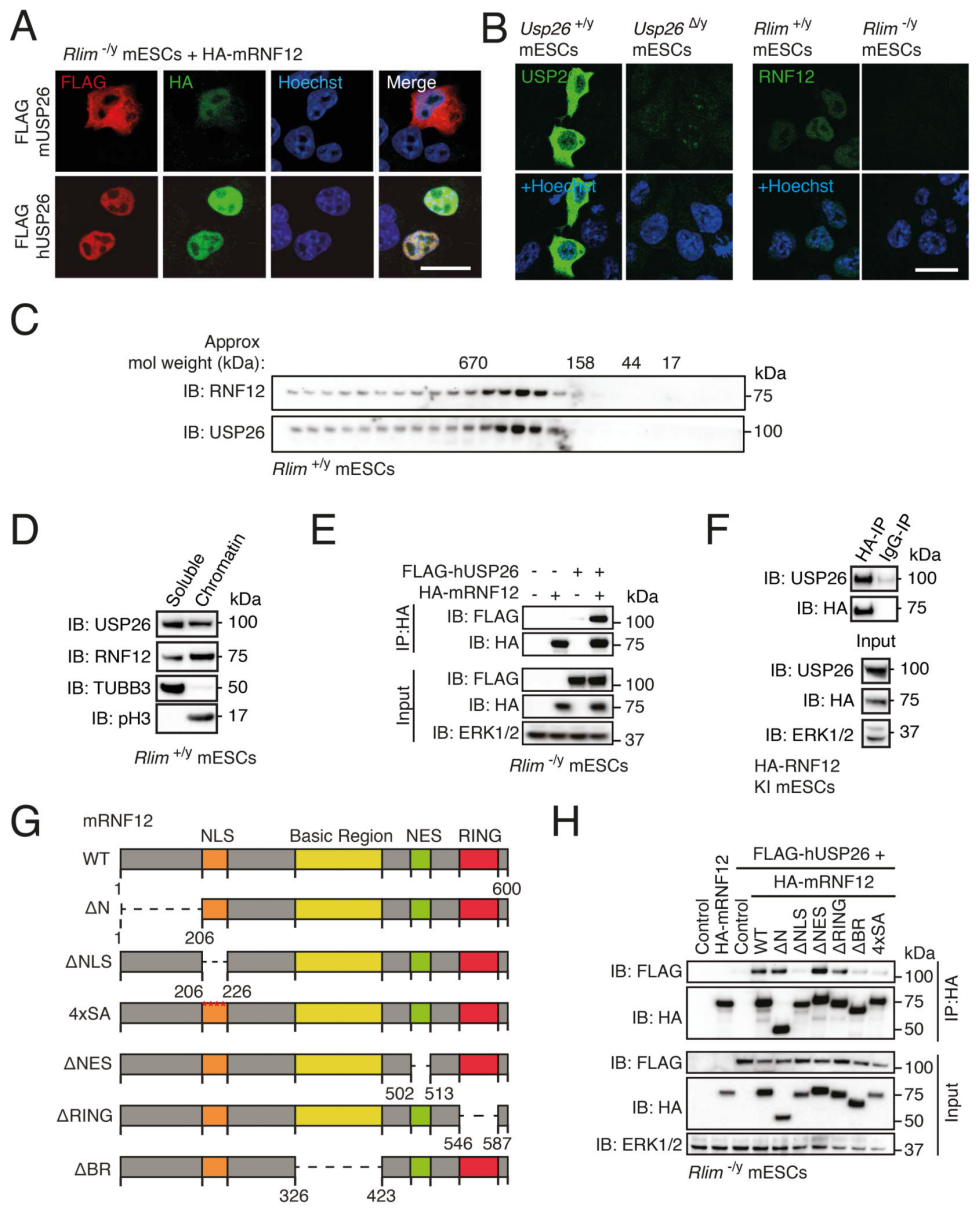
USP26 colocalizes with RNF12 and interacts with the RNF12 basic region. (**A**) Immunofluorescence localization of FLAG-tagged human USP26 (FLAG-hUSP26) and FLAG-tagged mouse USP26 (FLAG-mUSP26) coexpressed with HA-mRNF12 in *Rlim^-/y^* mESCs. Nuclei were stained with Hoechst. Scale bar, 20 μm. Data are representative of n = 3 independent experiments. (**B**) Immunofluorescence localization of endogenous USP26 in *Usp26^+/y^* and *Usp26^Δ/y^* mESCs and RNF12 in *Rlim^+/y^* and *Rlim^-/y^* mESCs. Nuclei were stained with Hoechst. Scale bar, 20 μm. Data are representative of n = 2 independent experiments. (**C**) Immunoblotting (IB) for USP26 and RNF12 in size-exclusion chromatography fractions of *Rlim^+/y^* mESC extracts. Data are representative of n = 3 independent experiments. (**D**) Immunoblotting for USP26 and RNF12 in *Rlim*^+/y^ mESCs fractionated into soluble cytoplasmic and nucleoplasmic (TUBB3) and chromatin-associated (phospho-Ser10 Histone H3, pH3) fractions. ERK1/2 is a loading control. Data are representative of n = 3 independent experiments. (**E**) Immunoblotting for FLAG and HA in HA immunoprecipitates (IP) from *Rlim^-/y^* mESCs expressing the indicated combinations of empty vector (-), FLAG-hUSP26, and HA-mRNF12. Data are representative of n = 3 independent experiments. (**F**) Immunoblotting for USP26, HA, and ERK1/2 in immunoprecipitates from endogenous HA-tagged RNF12 knock-in (HA-RNF12 KI) mESCs. Immunoprecipitation with IgG is a negative control. Data are representative of n = 3 independent experiments. (**G**) mRNF12 deletion mutants used for mapping the interaction with hUSP26. In the 4xSA mutant, Ser residues at positions 212, 214, 227, and 229 were mutated to Ala. (**H**) Immunoblotting for FLAG and HA in HA immunoprecipitates from *Rlim^-/y^* mESCs expressing empty vector (control) or FLAG-hUSP26 with the indicated HA-tagged mRNF12 deletion mutants. Data are representative of n = 3 independent experiments.

Our data indicate that transcriptional induction of *Usp26* is a major function of the RNF12-REX1 pathway. We therefore hypothesized that USP26 plays a role in regulating RNF12-dependent ubiquitin signaling. Because mouse and human USP26 are poorly conserved (37% identity and 55% similarity at the amino acid level), we first explored whether mouse and human USP26 colocalize and/or interact with mRNF12. In *Rlim^-/y^* mESCs reconstituted with HA-tagged mRNF12, FLAG-tagged mouse USP26 (mUSP26) localized to the cytoplasm with a proportion localized to the nucleus, whereas tagged human USP26 (hUSP26) and HA-mRNF12 were predominantly localized to the nucleus (Fig. 2A). Endogenous USP26 displayed a similar subcellular localization as transgenically expressed tagged mUSP26 (Fig. 2B). The specificity of endogenous USP26 immunofluorescence was confirmed using *Usp26^Δ/y^* mESCs generated by CRISPR-Cas9 genome editing (Fig. 2B, fig. S2, A to C). Gel filtration indicated that endogenous RNF12 and USP26 from mESCs co-eluted at a predicted molecular weight of ~200-500kDa (Fig. 2C) and, furthermore, co-fractionated with chromatin (Fig. 2D). These data suggest that RNF12 and USP26 may form a complex in mESCs. Indeed, co-immunoprecipitation revealed that FLAG-hUSP26 specifically interacted with HA-mRNF12 (Fig. 2E). Similarly, endogenous USP26 coimmunoprecipitated with mRNF12 in knock-in mESCs in which N-terminally HA-tagged mRNF12 was expressed from the endogenous locus (Fig. 2F). These data confirm that RNF12 and USP26 indeed colocalize and form a complex in mESCs.

To define the RNF12 motif(s) responsible for interaction with USP26, we performed coimmunoprecipitation analyses using hUSP26 and various mRNF12 deletion constructs lacking specific functional regions (Fig. 2G). The N-terminal region, the catalytic RING domain, and the nuclear export signal (NES) were dispensable for hUSP26 interaction (Fig. 2H). Deletion of the mRNF12 nuclear localization signal (NLS) or mutation of the NLS phosphorylation sites (4xSA) disrupted the interaction with hUSP26 (Fig. 2G), presumably because these mutants were mislocalized to the cytosol and therefore segregated from nuclear hUSP26 (fig. S3) (17, 30). However, the mRNF12 basic region (BR) deletion mutant, which colocalized to the nucleus with hUSP26 (fig. S1), failed to interact with hUSP26 (Fig. 2H). We therefore conclude that USP26 formed a complex with RNF12 by interacting with the BR.

### RNF12 autoubiquitylation leads to rapid proteasomal degradation

**Fig. 3:**
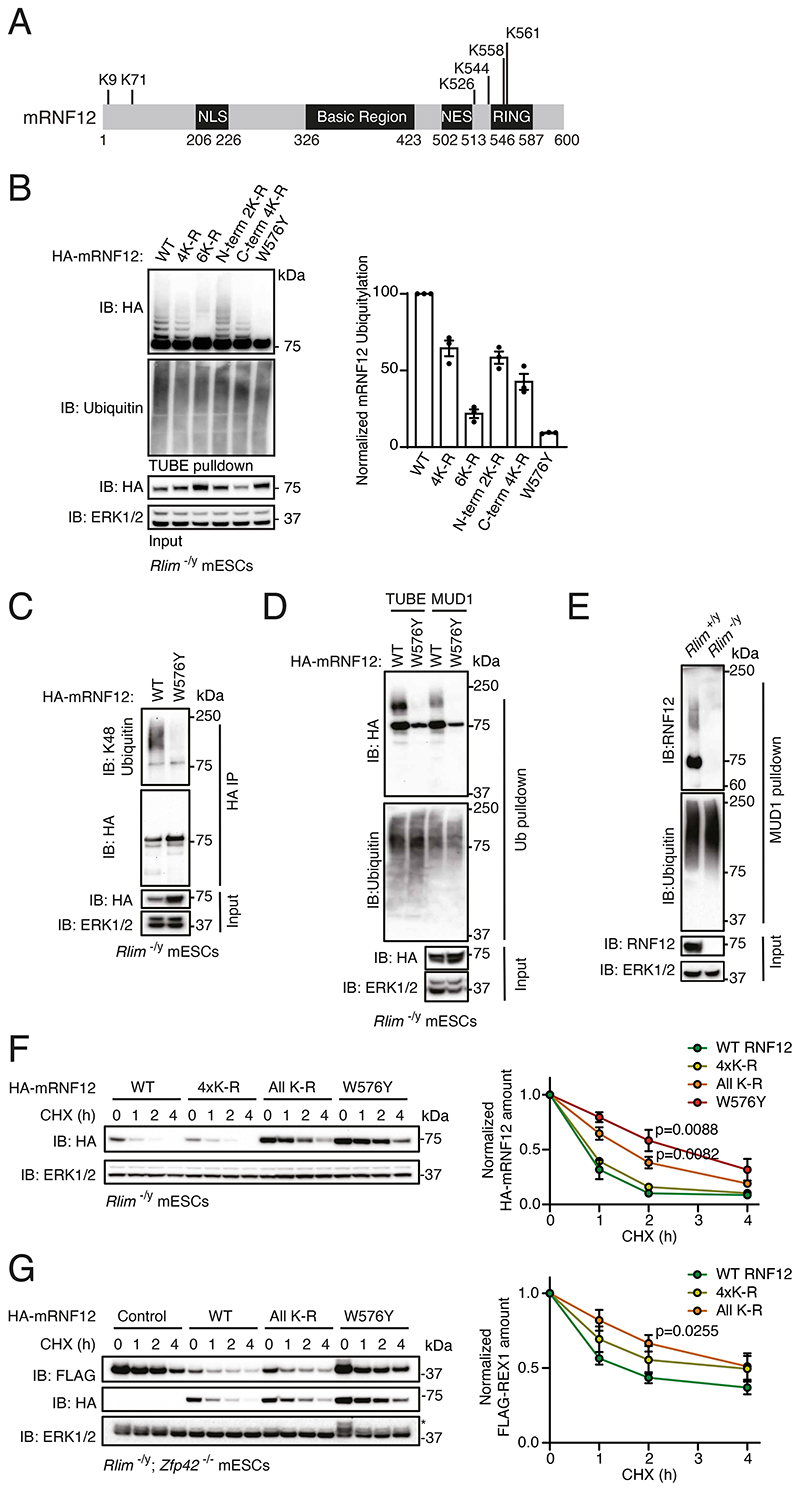
RNF12 autoubiquitylation leads to rapid proteasomal degradation. (**A**) mRNF12 domain structure with positions of all 6 Lys residues indicated. (**B**) TUBE pulldown from *Rlim^-/y^* mESCs expressing HA-mRNF12 WT, K71/526/544/558R (4xK-R), K9/71/526/544/558/561R (All K-R), K9/71R (N-term 2xK-R), K526/544/558/561R (C-term 4xK-R), or catalytic mutant (W576Y). HA-mRNF12, and ubiquitin were detected by immunoblotting (IB) and quantified. ERK1/2 is a loading control. Data represented as mean ± S.E.M. (**C**) HA immunoprecipitates (IP) from *Rlim^-/y^* mESCs expressing HA-mRNF12 WT or W576Y. K48-linked ubiquitin, HA, and ERK1/2 were detected by immunoblotting. (**D**) Ubiquitin (Ub) was pulled down from *Rlim^-/y^* mESCs expressing HA-mRNF12 WT or W576Y using TUBE or MUD1 ubiquitin-binding elements. HA, ubiquitin, and ERK1/2 were detected by immunoblotting. (**E**) MUD1 pulldown from *Rlim^+/y^* or *Rlim^-/y^* mESCs. RNF12, ubiquitin, and ERK1/2 were detected by immunoblotting. (**F**) *Rlim^-/y^* mESCs expressing HA-mRNF12 WT, K71/526/544/558R (4xK-R), K9/71/526/544/558/561R (All K-R), or catalytic mutant (W576Y) were treated with cycloheximide (CHX) for the indicated times. HA and ERK1/2 were detected by immunoblotting and quantified. Data represented as mean ± S.E.M. Statistical significance compared to HA-mRNF12 WT was determined by paired T-test; confidence level 95%. (**G**) *Rlim^-/y^; Zfp42^-/-^* mESCs expressing FLAG-REX1 and either empty vector or HA-mRNF12 WT, all K-R, or catalytic mutant (W576Y) were treated with CHX for the indicated times. HA, FLAG, and ERK1/2 were detected by immunoblotting and quantified. The asterisk marks a residual FLAG-REX1 signal from previous use of the blot. Data represented as mean ± S.E.M. Statistical significance compared to HA-mRNF12 WT was determined by paired T-test; confidence level 95%. For all panels, n = 3 independent experiments.

We next sought to determine the function of RNF12-USP26 complex formation. An important facet of the ubiquitin system with important regulatory consequences is “self-destruction,” in which E3 ubiquitin ligases autoubiquitylate (31). Therefore, we explored the possibility that USP26 recruitment counteracts RNF12 autoubiquitylation and proteasomal degradation. RNF12 has been reported to be autoubiquitylated (13), and previous mass spectrometry studies (32–34) suggest that RNF12 can be ubiquitylated on all six possible acceptor lysine residues in vivo (Fig. 3A; www.phosphosite.org). Indeed, WT mRNF12 was ubiquitylated in mESCs, as determined by tandem-ubiquitin binding element (TUBE) enrichment of ubiquitylated proteins followed by immunoblotting (Fig. 3B). Although mRNF12 ubiquitylation was not affected by mutation of 4 of the 6 potential acceptor lysine residues to arginine (K71/526/544/558R = 4xK-R; Fig. 3B), ubiquitylation was lost when all potential acceptor lysine residues were mutated (All K-R; Fig. 3B). Mutation of only the 2 N-terminal Lys residues (K9/71R = N-term 2xK-R) or the 4 C-terminal Lys residues (K526/544/558/561R = C-term 4xK-R) partially disrupted mRNF12 ubiquitylation, with greater disruption observed following mutation of 4xC-terminal Lys residues (Fig. 3B). mRNF12 ubiquitylation was abolished in the catalytically inactive mutant (W576Y; Fig. 3B), consistent with previous findings that RNF12 undergoes autoubiquitylation (13).

We investigated the ubiquitin chain linkage topology associated with RNF12 auto-ubiquitylation and found that RNF12 autoubiquitylation generated K48-linked ubiquitin chains, which are primarily associated with targeting for proteasomal degradation (35), as determined by probing HA-mRNF12 immunoprecipitates with a K48-specific antibody (Fig. 3C) or using MUD1 ubiquitin binding elements that are selective for K48-linked ubiquitin chains (36) (Fig. 3D). Endogenous RNF12 from mESCs was also modified by K48-linked ubiquitin chains, as confirmed by MUD1 ubiquitin binding elements (Fig. 3E). Furthermore, linkage-specific antibodies suggested that mRNF12 was modified by K48-linked ubiquitin but was not substantially modified by K11- or K63-linked chains (fig. S4). However, it should be noted that RNF12 may be modified by trace K11- or K63-linked chains or other types of ubiquitin linkage. As expected, RNF12 autoubiquitylation led to proteasomal degradation, because WT mRNF12 was degraded more rapidly than a non-ubiquitylatable mRNF12 mutant (All K-R) or a mRNF12 catalytic mutant (W576Y; Fig. 3F). mRNF12 All K-R retained comparable catalytic E3 ubiquitin ligase activity to WT mRNF12, as determined by the ability to promote REX1 degradation (Fig. 3G). In summary, these data demonstrate the importance of RNF12 autoubiquitylation in controlling protein stability and suggest a potential function for USP26 recruitment in modulating this process.

### USP26 inhibits RNF12 autoubiquitylation and suppresses RNF12 proteasomal degradation

**Fig. 4:**
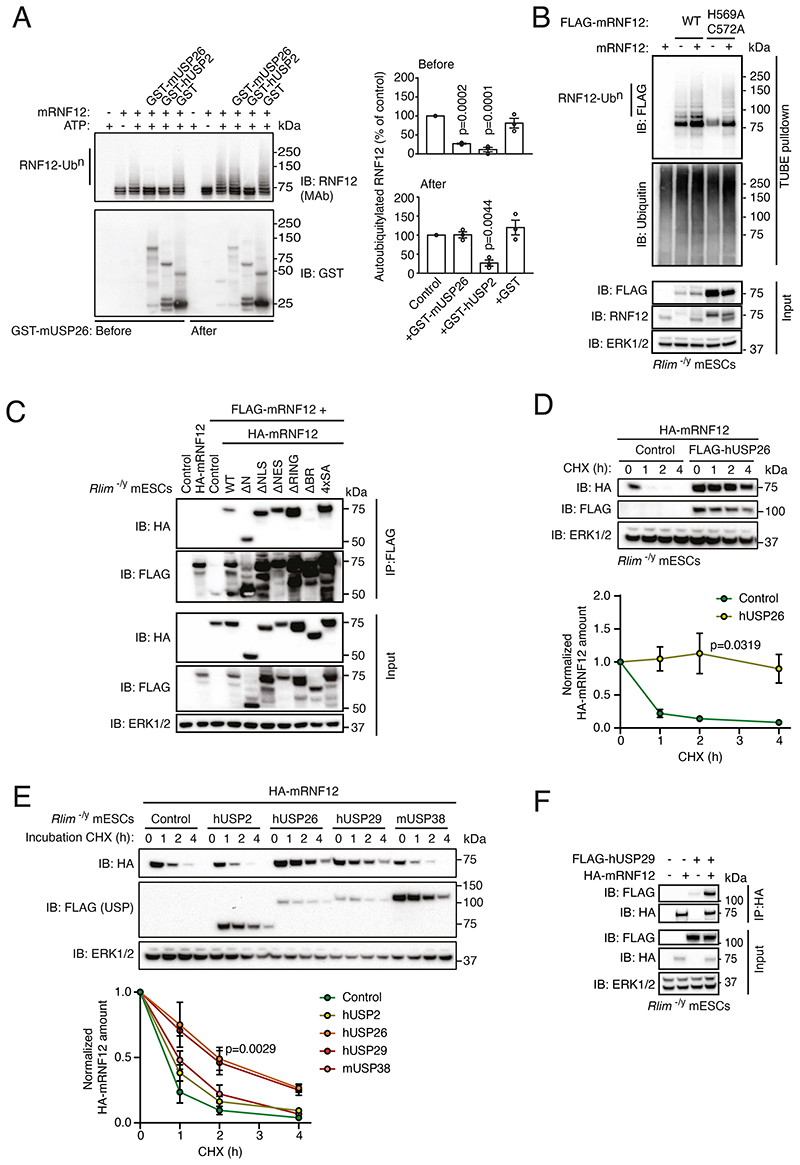
Interaction with USP26 inhibits RNF12 autoubiquitylation and suppresses proteasomal degradation. (**A**) mRNF12 in vitro autoubiquitylation in the presence of GST-mUSP26, GST-hUSP2, or GST added before or after mRNF12 autoubiquitylation. RNF12 and GST proteins were detected by immunoblotting (IB), and autoubiquitylated RNF12-Ub^n^ was quantified. Data represented as mean ± S.E.M. Statistical significance compared to control was determined by paired T-test; confidence level 95%. (**B**) TUBE-mediated ubiquitin pulldown from *Rlim^-/y^* mESCs expressing the indicated combinations of empty vector or mRNF12 WT and FLAG-mRNF12 WT or H569A/C572A. FLAG, and ubiquitin were detected by immunoblotting. ERK1/2 is a loading control. (**C**) FLAG immunoprecipitates (IP) from *Rlim^-/y^* mESCs expressing the indicated combinations of empty vector (Control), FLAG-mRNF12 WT and HA-mRNF12 WT (1-600), Δ1-206 (ΔN), Δ206-226 (ΔNLS), Δ502-513 (ΔNES), Δ546-587 (ΔRING), Δ326-423 (ΔBR), or S212l214l227l229A (4xSA). FLAG, HA, and ERK1/2 were detected by immunoblotting. (**D**) *Rlim^-/y^* mESCs expressing empty vector (Control) or FLAG-hUSP26 and HA-mRNF12 WT and treated with cycloheximide (CHX) for the indicated times. FLAG, HA, and ERK1/2 were detected by immunoblotting and quantified. Data represented as mean ± S.E.M. Statistical significance compared to control was determined by paired T-test; confidence level 95%. (**E**) *Rlim^-/y^* mESCs expressing HA-mRNF12 plus empty vector (Control) or FLAG-hUSP2, hUSP26, hUSP29, or mUSP38 and treated with CHX for the indicated times. FLAG-USP26, HA-RNF12, and ERK1/2 were detected by immunoblotting and quantified. Data represented as mean ± S.E.M. Statistical significance compared to control was determined by paired T-test; confidence level 95%. (**F**) HA immunoprecipitates from *Rlim^-/y^* mESCs expressing the indicated combinations of empty vector (-), FLAG-hUSP29, and HA-mRNF12. FLAG, HA, and ERK1/2 were detected by immunoblotting. For all panels, data are representative of n = 3 independent experiments.

Our results suggested that RNF12 specifically complexes with USP26, which we hypothesize may modulate RNF12 autoubiquitylation. In order to directly test this prediction, we incubated purified recombinant mRNF12 and mUSP26 and performed an in vitro ubiquitylation reaction. Autoubiquitylation of mRNF12 was clearly observed in this assay, as evidenced by the appearance of mRNF12 ubiquitylated bands (Fig. 4A). The addition of mUSP26 or the broad-specificity deubiquitylase hUSP2 before initiating the autoubiquitylation reaction prevented mRNF12 autoubiquitylation (Fig. 4A), suggesting that USP26 may catalyze deubiquitylation of autoubiquitylated RNF12. However, in contrast to hUSP2, addition of mUSP26 following completion of the mRNF12 autoubiquitylation reaction failed to remove mature ubiquitin chains (Fig. 4A). These results indicate that although USP26 inhibits RNF12 autoubiquitylation, USP26 appears to act at least in part either by removing nascent RNF12-linked ubiquitin chains or by interfering with RNF12 autoubiquitylation.

We next explored the mechanisms by which USP26 might interfere with RNF12 autoubiquitylation. Because RING E3 ubiquitin ligases frequently self-associate and autoubiquitylate in trans, we hypothesized that USP26 recruitment to RNF12 may disrupt this process. We previously showed that hRNF12 self-associates (*10*), which prompted us to determine whether RNF12 autoubiquitylates in trans. FLAG-mRNF12 WT was autoubiquitylated when expressed in RNF12 knockout (*Rlim^-/y^*) mESCs with or without untagged mRNF12 (Fig. 4B). When a FLAG-mRNF12 catalytic mutant (H569A/C572A) was expressed alone, no autoubiquitylation was detected (Fig. 4B), but when untagged WT mRNF12 was expressed with FLAG-mRNF12 H569A/C572A, the FLAG-mRNF12 H569A/C572A catalytic mutant was ubiquitylated (Fig. 4B). RNF12 ubiquitylation was largely dependent on its own catalytic activity, confirming that RNF12 can autoubiquitylate in trans, but we cannot rule out the possibility that RNF12 is also capable of autoubiquitylation in cis. Given that USP26 inhibited RNF12 autoubiquitylation and that RNF12 self-association facilitated its autoubiquitylation. we investigated whether USP26 might interfere with RNF12 self-association. We therefore defined the regions of RNF12 that were required for self-association by performing coimmunoprecipitation experiments with FLAG-tagged full-length mRNF12 and various HA-tagged deletion mutants (Fig. 4C). mRNF12 self-association was mediated by the basic region (BR), which was also required for the interaction with hUSP26 (Fig. 2H). This suggests that the interaction with USP26 may sterically hinder RNF12 self-association, thereby preventing autoubiquitylation. This is in addition to the potential for direct reversal of RNF12 autoubiquitylation by the intrinsic deubiquitylase activity of USP26.

Because RNF12 autoubiquitylation consists of K48-linked degradative ubiquitin chains that drive proteasomal degradation, we investigated whether inhibition of RNF12 autoubiquitylation by USP26 promoted RNF12 stabilization in cells. To this end, we reconstituted RNF12 signaling in *Rlim^-/y^* mESCs, which lack endogenous RNF12 and produce little USP26 (Fig. 1D), by selectively expressing HA-mRNF12 and/or FLAG-hUSP26. We confirmed that HA-mRNF12 had a relatively short half-life in mESCs due to autoubiquitylation and proteasomal degradation (Fig. 4D). However, expression of FLAG-hUSP26 stabilized HA-mRNF12 over a 4 h time course (Fig. 4D). This depended on proteasomal degradation, because mRNF12 was stabilized by hUSP26 or by the proteasome inhibitor MG132 (fig. S5A). RNF12 stabilization was specific to hUSP26 and its close relative hUSP29 (Fig. 4E), because expression of the more distantly related mUSP38 or hUSP2 did not stabilize RNF12 in mESCs (Fig. 4E). Furthermore, similar to hUSP26, hUSP29 was also capable of interacting with mRNF12 (Fig. 4F), consistent with its effect on RNF12 stability. In these experiments, hUSP26 was expressed at amounts similar to endogenous mUSP26 (fig. S5B; compare USP26 staining for endogenous and transgenically expressed mUSP26, and FLAG staining for mUSP26 and hUSP26; note that FLAG-hUSP26 is not detected by mUSP26 antibody), and hUSP26, hUSP29 and mUSP38 all colocalized with mRNF12 in the nucleus (fig. S5C). However, although hUSP2 is a broad-specificity deubiquitylase that deubiquitylates RNF12 in vitro (Fig. 4A), hUSP2 and mRNF12 did not colocalize in the nucleus (fig. S5C), which explains why hUSP2 did not stabilize mRNF12 in cells (Fig. 4E). These data indicate that USP26 and the closely related USP29 specifically suppress RNF12 autoubiquitylation and proteasomal degradation. Collectively, our results thus far reveal that *Usp26* is transcriptionally induced by RNF12-dependent degradation of REX1, leading to the formation of a RNF12-USP26 complex that stabilizes RNF12.

### The RNF12-USP26 signaling axis operates specifically in the testes

**Fig. 5:**
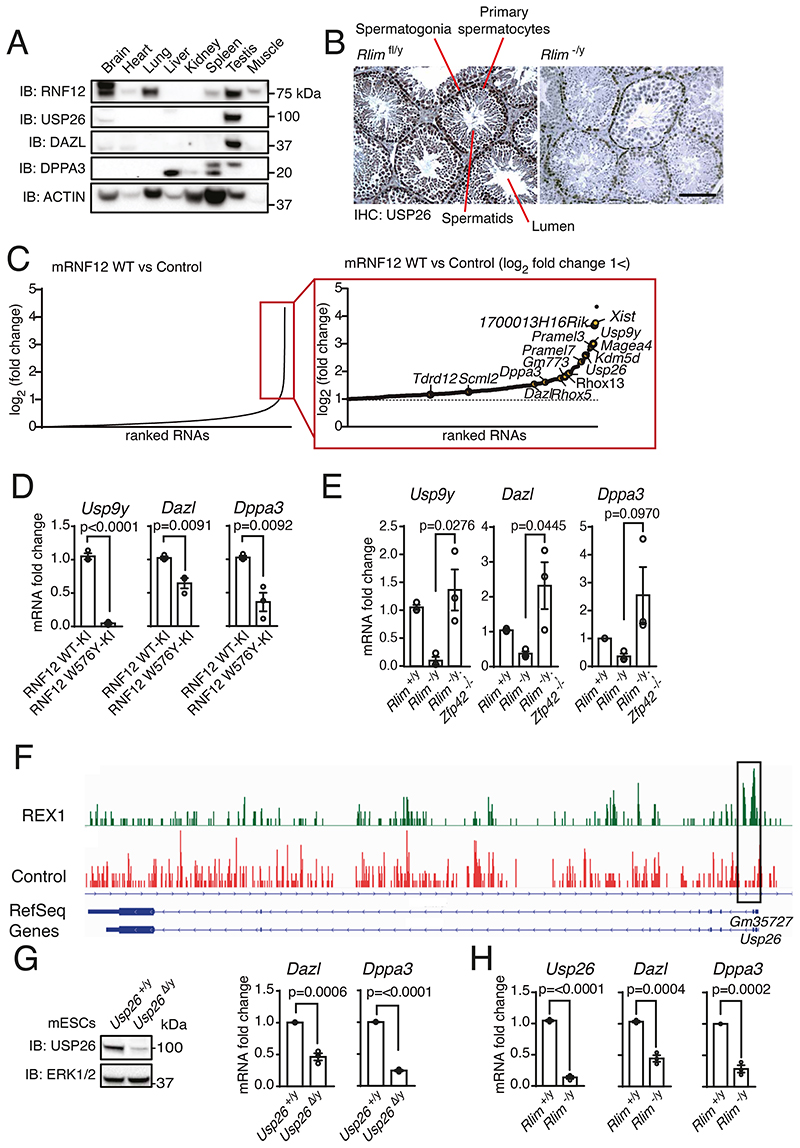
The RNF12-REX1-USP26 signaling axis operates in testes and promotes expression of germ cell–specific genes. (**A**) RNF12, USP26, DAZL, and DPPA3 were detected in the indicated mouse tissues by immunoblotting (IB). Actin is a loading control. Data are representative of n = 3 independent experiments. (**B**) USP26 immunohistochemistry of mouse testes sections from *Rlim^fl/y^* and *Rlim^-/y^* littermates. Scale bar, 100 μm. Data are representative of n = 2 animals of each genotype. (**C**) RNA-sequencing (RNA-SEQ) data from *Rlim^-/y^* mESCs expressing empty vector (Control) or WT-mRNF12 *(17)*. RNAs were ranked according to fold-change increase in expression. The magnified view of the boxed area shows RNAs for which expression increased >2-fold in *Rlim^-/y^* mESCs expressing mRNF12, with RNAs implicated in reproduction or germ cell development highlighted. (**D and E**) Quantification of *Usp9y, Dazl*, and *Dppa3* mRNAs in RNF12 WT-KI and W576Y-KI mESCs (D) and in *Rlim*^-/y^, *Rlim*^-/y^ and *Rlim*^✓y^; *Zfp42^-/-^* mESCs (E) by qRT-PCR. Data represented as mean ± S.E.M. n = 3 independent experiments. Statistical significance was determined by paired T-test; confidence level 95%. (**F**) REX1 and control chromatin immunoprecipitation followed by DNA sequencing peaks for the *Usp26* gene. REX1-enriched regions near the *Usp26* transcriptional start site are highlighted. (**G**) *Usp26^+/y^* and *Usp26*^Δ/y^ mESCs were cultured in 2i conditions. USP26 and ERK1/2 (loading control) were detected by immunoblotting, and *Dazl* and *Dppa3* mRNAs were quantified by qRT-PCR. Data represented as mean ± S.E.M. n = 3 independent experiments. Statistical significance was determined by paired T-test; confidence level 95%. (**H**) Quantification of the indicated mRNAs in *Rlim^+/y^* and *Rlim*^-/y in 2i^ mESCs by qRT-PCR. Data represented as mean ± S.E.M. n = 3 independent experiments. Statistical significance was determined by paired T-test; confidence level 95%.

We next investigated the physiological function of the RNF12-USP26 signaling axis. RNF12 is required for normal spermatogenesis (9), a process in which USP26 has also been implicated (24–28). Furthermore, *USP26* variants have been associated with azoospermia in various fertility disorders, including Sertoli-cell only syndrome (24, 25, 37). To explore the possibility that RNF12-USP26 signaling may contribute to spermatogenesis, we first sought to confirm the tissue distribution of RNF12 and USP26. As shown previously in mouse tissues, RNF12 protein was most abundant in adult brain, lung, and testes (17), whereas USP26 was largely restricted to mESCs and testes in a selection of somatic tissues (24) (Fig. 5A). USP26 was observed in primary spermatocytes and spermatids, and spermatogonia, the precursors of spermatocytes and spermatids, in the testes of control adult *Rlim^fl/y^* mice. USP26 was reduced in primary spermatocytes and spermatids of adult *Rlim^-/y^* mice but unchanged in spermatogonia (Fig. 5B).

This suggests that USP26 abundance does not depend on the RNF12-REX1 axis in spermatogenic stem cells but does depend on RNF12-REX1 in their progeny. These data confirm that the RNF12-USP26 signaling axis operates specifically in the mouse testes, raising the possibility that it may play an important function in this context.

### RNF12-REX1-USP26 signaling regulates the expression of gametogenesis genes

In order to identify potential molecular functions of the RNF12-USP26 axis that may be relevant to the biology of male reproduction and fertility, we analyzed our previously reported RNA-SEQ dataset for RNF12-dependent mRNAs (17). Amongst mRNAs that were induced by WT mRNF12 in mESCs (Fig. 5C), Gene Ontology (GO) term analysis identified enrichment of transcripts relating to metabolism, development, and differentiation (see data file S1 for a full list). However, the RNF12-dependent transcriptional signature in mESCs was also significantly enriched for genes associated with reproduction and gametogenesis (Fig. 5C; data file S1, Table S1). This included *Usp26* itself*, Dazl*, which encodes an RNA-binding protein that is essential for gametogenesis in both males and females (18), *Usp9y*, a Y-linked gene encoding a deubiquitylase associated with male infertility (19) and *Dppa3*, a primordial germ cell (PGC)-specific protein (20) (Fig. 5C). These data suggest that the RNF12-USP26 axis may function to promote the expression of genes that are required for gametogenesis and germ cell development, consistent with a functional role for the RNF12-USP26 axis in the testes.

These data prompted us to explore the mechanism by which RNF12 controls the expression of genes associated with gametogenesis. Initially, we sought to confirm that *Dazl, Usp9y*, and *Dppa3* were induced by RNF12 E3 ubiquitin ligase activity. We again employed control WT RNF12 knock-in mESCs (WT-KI) and RNF12 catalytic mutant knock-in mESCs (W576Y-KI) (17), in which endogenous RNF12 substrates, including REX1, accumulate (Fig. 1B). As expected, *Dazl, Usp9y*, and *Dppa3* were expressed in control RNF12 WT-KI mESCs but reduced in RNF12 W576Y-KI mESCs (Fig. 5D), confirming that RNF12 E3 ubiquitin ligase activity is critical for the expression of these genes. We then determined whether REX1 was the relevant RNF12 substrate driving the expression of key gametogenesis genes. Similar to *Usp26* (Fig. 1E), *Dazl, Usp9y*, and *Dppa3* mRNAs were suppressed by loss of RNF12 *(Rlim^-/y^)* but restored in RNF12;REX1 double-knockout mESCs *(Rlim^-/y^; Zfp42^-/-^)* (Fig. 5E). Furthermore, REX1 ChIP-SEQ data indicated that REX1 was directly recruited to the *Usp26* promoter (Fig. 5F) and the promoters of other RNF12-REX1-dependent gametogenesis genes (fig. S6). Taken together, our results indicate that RNF12, by ubiquitylating and thereby promoting degradation of the transcriptional repressor REX1, promotes a gene expression program that is associated with gametogenesis.

We next set out to address whether endogenous USP26 also promotes expression of gametogenesis genes. Because USP26 is produced in only a small percentage of mESCs when cultured in LIF-FCS (fig. S5B), we employed mESCs cultured under 2i conditions, in the presence of inhibitors of the kinases MEK1 and MEK2 (MEK1/2) and GSK3, which maintain them in the naïve “ground state”. To explore the function of endogenous USP26 in 2i mESCs, we employed *Usp26^Δ/y^* mESCs (fig. S2, A to C) and cultured the cells under 2i conditions. In this context, reduction of USP26 (*Usp26*^Δ/y^) (Fig. 5G; note residual USP26 expression in *Usp26*^Δ/y^ mESCs) or loss of RNF12 *(Rlim^-/y^)* (Fig. 5H) led to reduction in *Dazl and Dppa3* mRNAs. Therefore, these data indicate that endogenous USP26 is required for efficient expression of RNF12-dependent gametogenesis genes.

### RNF12 signaling promotes differentiation of mouse primordial germ cell-like cells

**Fig. 6:**
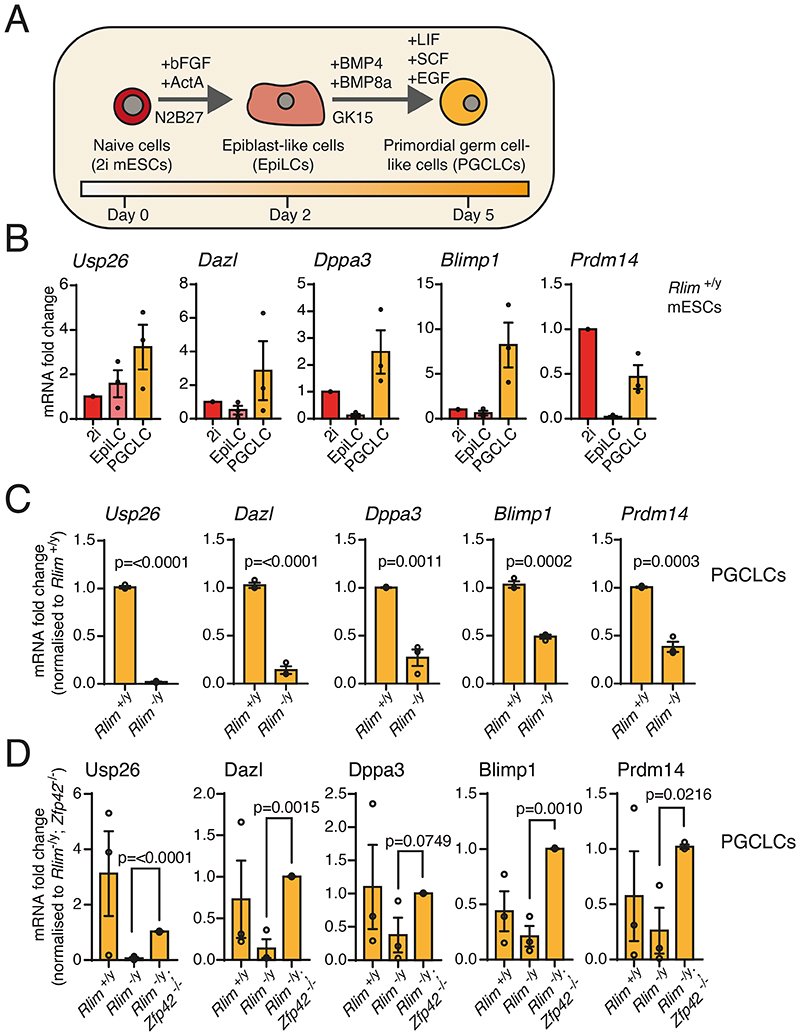
RNF12 is required for efficient germ cell differentiation in vitro. (**A**) Scheme for differentiating 2i mESCs to epiblast-like cells (EpiLCs) and primordial germ cell–like cells (PGCLCs). (**B**) Quantification of the indicated mRNAs in *Rlim^+/y^* 2i mESCs, EpiLCs, and PGCLCs by qRT-PCR. Data represented as mean ± S.E.M. (**C**) Quantification of the indicated mRNAs in *Rlim^+/y^* and *Rlim^-/y^* 2i mESCs differentiated to PGCLCs. Data was normalized to *Rlim^+/y^* mESCs and represented as mean ± S.E.M. Statistical significance was determined by paired T-test; confidence level 95%. (**D**) Quantification of the indicated mRNAs in *Rlim^+/y^, Rlim^-/y^* and *Rlim^-/y^; Zfp42^-/-^* mESCs differentiated to PGCLCs. Data was normalized to *Rlim^-/y^; Zfp42^-/-^* mESCs and represented as mean ± S.E.M. Statistical significance was determined by paired T-test; confidence level 95%. For all panels, n = 3 independent experiments.

Our results thus far indicated that the RNF12-USP26 axis specifically operates in the testes and promotes the expression of genes associated with gametogenesis. Thus, we sought to address the function of the RNF12 pathway in germ cell development in vitro. To this end, we employed an established protocol for differentiation of primordial germ cell-like cells (PGCLCs) from mESCs (38) in which 2i mESCs are differentiated first to epiblast-like cells (EpiLCs) and then to PGCLCs (Fig. 6A). mESCs efficiently differentiated to PGCLCs by this method, as assessed by the induction of PGCLC markers *Dazl, Dppa3, Blimp1*, and *Prdm14* (Fig. 6B). *Usp26* expression was also induced during PGCLC differentiation (Fig. 6B). RNF12 knockout reduced the efficiency of PGCLC differentiation (Fig. 6C), whereas RNF12;REX1 double knockout restored the induction of PGCLC markers (Fig. 6D). Notably, *Usp26* expression was largely dependent on RNF12 in these experiments (Fig. 6C and D), confirming that the RNF12-USP26 axis is operational in the context of PGCLC differentiation. In summary, our data indicate that the RNF12 pathway plays a crucial role in promoting germ cell differentiation in vitro.

### Disrupted RNF12-USP26 signaling contributes to Tonne-Kalscheuer Syndrome (TOKAS) and azoospermia

**Fig. 7:**
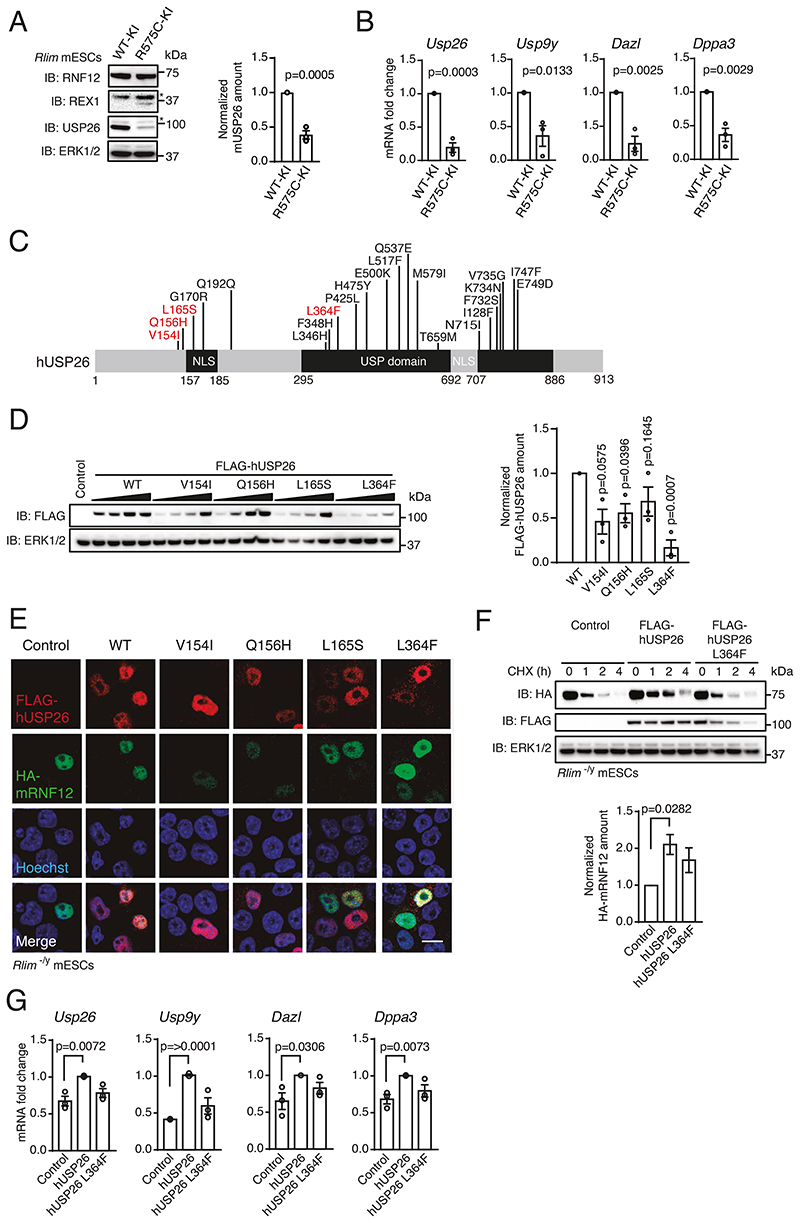
*RLIM* and *USP26* variants associated with human genetic disorders functionally disrupt the RNF12-USP26 signaling axis. (**A**) RNF12, REX1, and USP26 in control RNF12 WT knock-in (WT-KI) and RNF12 TOKAS mutant knock-in (R575C-KI) mESCs were detected by immunoblotting (IB) and quantified. ERK1/2 is a loading control. Asterisk indicates a non-specific band. Data represented as mean ± S.E.M. Statistical significance was determined by paired T-test; confidence level 95%. (**B**) Quantification of the indicated mRNAs in RNF12 WT-KI and R575C-KI mESCs by qRT-PCR. Data represented as mean ± S.E.M. Statistical significance was determined by paired T-test; confidence level 95%. (**C**) hUSP26 showing variants associated with azoospermia. Prioritized frequently reported variants used for subsequent experiments are highlighted in red. (**D**) Immunoblotting for FLAG in *Rlim^-/y^* mESCs expressing empty vector or increasing amounts of FLAG-hUSP26 WT or the indicated azoospermia variants. Data represented as mean ± S.E.M. Statistical significance was determined by paired T-test; confidence level 95%. (**E**) Representative immunofluorescence images of HA-mRNF12 and FLAG-hUSP26 in *Rlim^-/y^* mESCs expressing HA-mRNF12 and FLAG-hUSP26 WT or indicated azoospermia variants. Nuclei were stained with Hoechst. Scale bar, 10 μm. (**F**) *Rlim^-/y^* mESCs expressing HA-mRNF12 and either empty vector or FLAG-hUSP26 WT or L364F were treated with cycloheximide (CHX) for the indicated times. FLAG-, HA, and ERK1/2 were detected by immunoblotting. Quantification shows 2 h samples only. Data represented as mean ± S.E.M. Statistical significance was determined by paired T-test; confidence level 95%. (**G**) Quantification of the indicated mRNAs in *Rlim^-/y^* mESCs expressing HA-mRNF12 and either empty vector or FLAG-hUSP26 WT or L364F by qRT-PCR. Data represented as mean ± S.E.M. Statistical significance was determined by paired T-test; confidence level 95%. For all panels, n = 3 independent experiments.

Finally, we investigated whether the RNF12-USP26 axis is dysregulated in human disease. Variants in *RLIM* that disrupt RNF12 E3 ubiquitin ligase activity cause TOKAS, a developmental disorder characterized by intellectual disability and syndromic anomalies including urogenital defects with hypogenitalism in affected males and fertility defects in heterozygous carrier females (6). To determine the impact of a TOKAS-associated RNF12 variant on the regulation and function of the RNF12-USP26 axis, we took advantage of knock-in mESCs harboring the mouse equivalent of the human RNF12 R599C variant identified from a TOKAS patient kindred (RNF12 R575C-KI) (10). Compared to control RNF12 knock-in mESCs (RNF12 WT-KI), RNF12 R575C-KI mESCs displayed impaired RNF12 E3 ubiquitin ligase activity and REX1 accumulation (10) (Fig. 7A). Accordingly, RNF12 R575C-KI mESCs displayed reduced USP26 (Fig. 7A), suggesting that RNF12 TOKAS variants may interfere with *Usp26* gene expression. Indeed, *Usp26* mRNA was diminished in RNF12 R575C-KI mESCs compared to control RNF12 WT-KI mESCs (Fig. 7B). RNF12 R575C-KI mESCs also displayed reduced *Usp9y, Dazl*, and *Dppa3* mRNAs (Fig. 7B). Our data therefore demonstrate that a RNF12 TOKAS variant disrupts expression of *Usp26* and other gametogenesis genes, suggesting that the RNF12-USP26 axis is likely impaired in patients harboring a disease-causing variant of RNF12.

*USP26* gene variants have been implicated in the azoospermia, such as that associated with sertoli-cell only syndrome (24, 25, 37). We therefore hypothesized that *USP26* variants identified from azoospermia patients may also lead to deregulation of the RNF12-USP26 axis and gametogenesis gene expression. *USP26* azoospermia variants result in amino acid substitutions largely clustered around a nuclear localization sequence (NLS) and within the USP catalytic domain (Fig. 7C), with one variant implicated in the disruption of catalytic activity (39). Thus, we prioritized a panel of hUSP26 variants (Fig. 7C) to investigate the effects of those amino acid substitutions on USP26 and RNF12 function. In most cases, hUSP26 variants exhibited reduced protein abundance when expressed in mESCs (Fig. 7D). However, all hUSP26 variants tested, including those surrounding the NLS, localized correctly to the nucleus (Fig. 7E). These data suggest that reduced protein abundance may be a pathogenic mechanism in fertility patients harboring *USP26* variants.

Our finding that *USP26* gene variants associated with fertility defects reduced protein expression in mESCs suggests that these variants may affect RNF12 deubiquitylation and stabilization. We addressed this possibility using USP26 L364F, a variant within the USP catalytic domain (Fig. 7C) that was very poorly expressed in mESCs (Fig. 7D). As shown previously, mRNF12 was rapidly degraded as a result of autoubiquitylation but was significantly stabilized by expression of WT hUSP26 compared to control (Fig. 7F). When expressed in equivalent amount to WT hUSP26, hUSP26 L364F also stabilized RNF12 to some extent, but the variant’s stabilization of mRNF12 was not statistically significant compared to control cells not expressing hUSP26 (Fig. 7F), indicating that USP26 azoospermia variants may interfere with USP26 function in preventing RNF12 autoubiquitylation and proteasomal degradation. Of note, we found that WT hUSP26 was a relatively long-lived protein, whereas the hUSP26 L364F variant was rapidly degraded (Fig. 7F), suggesting that impaired stability may explain the reduced protein amounts observed for USP26 azoospermia variants.

Lastly, we explored the impact of the hUSP26 L364F azoospermia-associated variant on RNF12-dependent regulation of gametogenesis gene expression. Expression of WT hUSP26 resulted in increased expression of *Usp26, Usp9y, Dazl*, and *Dppa3* (Fig. 7G), whereas induction of these genes by the azoospermia-associated USP26 L364F variant was not significantly different from control samples (Fig. 7G). Taken together, our data indicate that the RNF12-USP26 axis promotes gametogenesis gene expression, and this is functionally disrupted by *RLIM* and *USP26* variants associated with TOKAS and azoospermia, respectively.

## Discussion

Ubiquitylation is critical for regulating many developmental processes and as such ubiquitylation components are mutationally disrupted in human developmental disorders. Here, we uncovered a ubiquitylation axis involving the RING-type E3 ubiquitin ligase RNF12 and the deubiquitylase USP26 that controls gametogenesis gene expression. RNF12 E3 ubiquitin ligase activity promoted *Usp26* gene transcription and an increase in USP26 protein, which stimulated complex formation between RNF12 and USP26, resulting RNF12 stabilization. This system created a feed-forward amplification loop that drove RNF12-dependent signaling and downstream transcription.

**Fig. 8:**
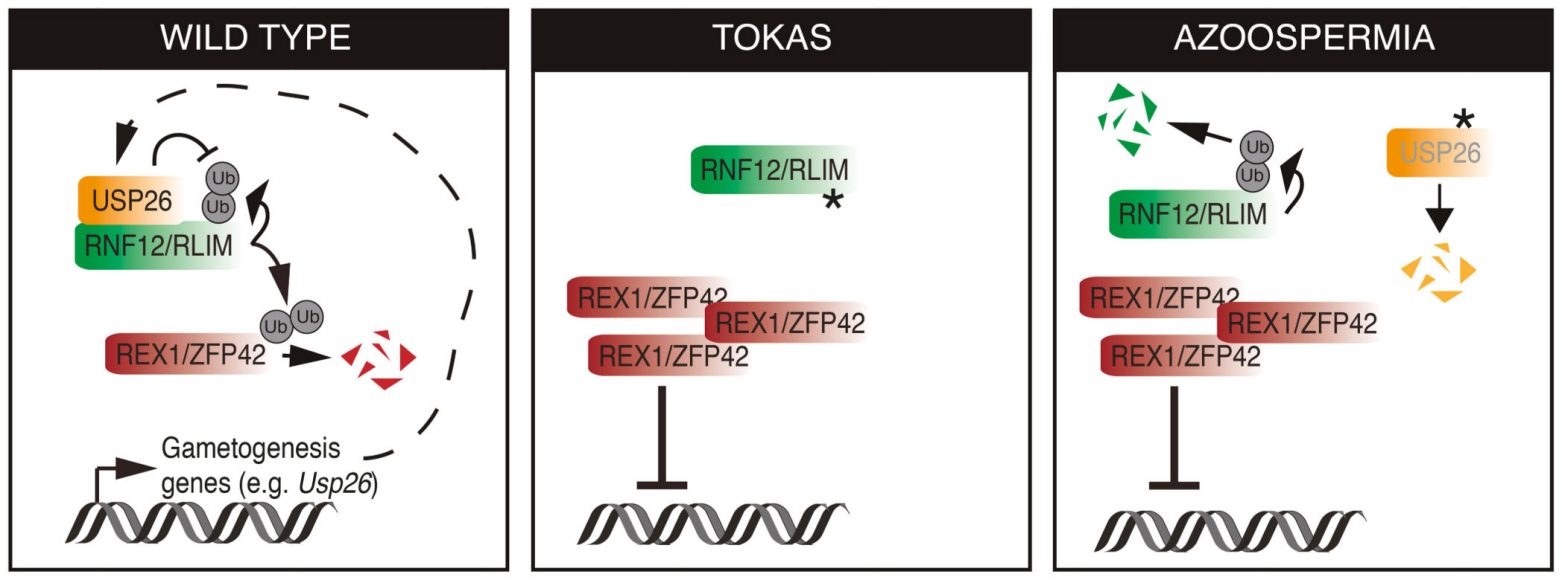
An RNF12-USP26 amplification loop promotes germ cell specification and is disrupted in human genetic disorders. In wild-type germline stem cells, RNF12 ubiquitylates REX1 to relieve transcriptional repression of gametogenesis genes, including *Usp26*. The deubiquitylase USP26 complexes with and stabilizes RNF12 by preventing RNF12 autoubiquitylation and/or deubiquitylating RNF12, which amplifies RNF12 signaling to drive gametogenesis gene expression. In Tonne-Kalscheuer syndrome (TOKAS), RNF12 variants disrupt catalytic activity, resulting in REX1 accumulation, transcriptional repression of gametogenesis genes, and disruption of USP26 feed-forward activation. In azoospermia, USP26 variants fail to protect RNF12 from autoubiquitylation, disrupting the feed-forward mechanism and maintaining transcriptional repression of gametogenesis genes.

Furthermore, we provided evidence that the amplification loop is of critical importance in human disease, because it is disrupted by *RLIM* and *USP26* variants that cause human genetic disorders. Thus, our results provide detailed molecular insight into the complex interplay within the ubiquitin system in the regulation of developmental processes and how this is dysregulated in disease (Fig. 8). Of particular interest is the fact that an E3 ubiquitin ligase employs transcriptional induction of a deubiquitylase to amplify its own function, which is an unprecedented molecular mechanism for activation of a ubiquitin signaling pathway. However, this fits with previous understanding of how cell fate decisions are executed and reinforced during development, which frequently involves amplification and negative feedback loops within the signaling and transcriptional machinery to confer robust cellular decision-making (40).

A key question that remains to be answered is the molecular mechanism by which USP26 stabilizes RNF12. Our data indicate that USP26 is recruited to RNF12, which may directly catalyze deubiquitylation and stabilization. However, the fact that USP26 engages RNF12 within the basic region that is involved both in catalysis and self-association for autoubiquitylation suggests that USP26 might function by a more complex mechanism whereby it disrupts RNF12 self-association —and potentially also RNF12 catalytic activity—to prevent auto-ubiquitylation. This is supported by our demonstration that recombinant USP26 inhibited RNF12 autoubiquitylation in vitro only when added prior to the autoubiquitylation reaction, suggesting that USP26 may operate independently of RNF12 catalytic activity, at least in part. Alternatively, USP26 may be able to remove only simple ubiquitin modifications as they are added to RNF12, and not the complex ubiquitin chains observed following the RNF12 autoubiquitylation reaction. This question can be resolved by the development of USP26 catalytically defective mutants and recombinant USP26 with verified deubiquitylase activity, which has thus far proven elusive.

Finally, we showed that the RNF12-USP26 axis operated specifically in the testes and was disrupted in human genetic disorders associated with genital abnormalities and/or infertility. In this regard, *USP26* variants are found in azoospermia patients, and *RLIM* variants cause TOKAS, which is characterized by syndromic features including urogenital abnormalities in affected males and fertility problems in carrier females. However, the specific role of the RNF12-USP26 axis in gametogenesis and fertility in vivo is yet to be determined. For example, it is not yet clear whether affected male TOKAS patients exhibit fertility defects, although recent research indicates that male RNF12 knockout (*Rlim^-/y^*) mice have a defect in spermiogenesis (9). Similarly, the role of USP26 in gametogenesis has not yet been defined, and there are conflicting data about whether USP26 is required for male fertility in mouse models (24–28). In this case, it appears likely that USP29, which is also present in emerging PGCLCs and testes and can stabilize RNF12, may compensate for the loss of USP26 function. Therefore, further research is required to determine the specific function of RNF12-USP26 in gametogenesis in vivo.

## Materials and Methods

### Cell culture

Male mouse embryonic stem cells (mESCs, CCE line) were originally from the laboratory of Janet Rossant, SickKids Research Institute, Toronto. These are authenticated by pluripotent embryonic stem cell marker expression analysis and confirmed mycoplasma negative. mESCs were cultured in ES-DMEM media (DMEM base, 5% knockout serum replacement (KO serum) (v/v), 2 mM glutamine, 0.1 mM minimum essential media (MEM) nonessential amino acids, 1 mM sodium pyruvate, 50 U/ml penicillin, 50 μg/ml streptomycin, 10% FCS (v/v), 0.1 mM β-mercaptoethanol; supplemented with 20 ng/mL LIF (Medical Research Council Protein Phosphorylation and Ubiquitin Unit Reagents and Services (MRC-PPU R&S)) on plates coated with 0.1% gelatin at 5% CO_2_ and 37°C.

### Primordial germ cell like-cell (PGCLC) differentiation

mESCs were cultured in 2i medium consisting of N2B27 media with 1% (v/v) B27 supplement, 0.5% (v/v) N2 supplement, 2 mM glutamine (all from Thermo Fisher Scientific), 0.1 mM β-mercaptoethanol (Sigma-Aldrich) and penicillin and streptomycin in 1:1 DMEM F12:Neurobasal medium (both from Thermo Fisher Scientific) supplemented with a cocktail of inhibitors (0.4 μM PD0325901 and 3 μM CHIR99021, Axon) and 20 ng/mL LIF (MRC-PPU R&S). Epiblast-like cell (EpiLC) differentiation was induced by plating 1x10^5^ 2i mESCs per well in a 0.1% gelatin coated 12-well plate. EpiLCs were kept in EpiLC medium consisting of N2B27 medium supplemented with 20 ng/ml Activin A and 12 ng/ml bFGF (both from Peprotech), and 1% knockout serum replacement (KSR) (Thermo Scientific) at 5% CO_2_ and 37°C for two days. EpiLCs were differentiated into primordial germ cell-like cells (PGCLCs) by plating 2x10^3^ EpiLCs per well in a low cell binding U-bottom 96-well plate in serum-free medium (GK15 medium consisting of GMEM (Thermo Fisher Scientific)) with 15% KSR, 0.1 mM NEAA, 1 mM sodium pyruvate, 0.1 mM β-mercaptoethanol, 100 U/ml penicillin, 0.1 mg/ml streptomycin, and 2 mM I-glutamine) in the presence of the cytokines BMP4 (500 ng/ml; R&D Systems), LIF (1,000 U/ml; Merck Millipore), SCF (100 ng/ml; R&D Systems), and EGF (50 ng/ml; R&D Systems). After three days in culture, PGCLC clusters were harvested for RNA extraction or immunofluorescence.

### Transfection and plasmids

mESCs were transfected using Lipofectamine LTX (Thermo Fisher Scientific) according to the manufacturer’s instructions. pCAGGS puro plasmids (table S2) were generated by the MRC-PPU R&S and verified by DNA sequencing (MRC-PPU DNA Sequencing & Services) using Applied Biosystems Big-Dye Ver 3.1 chemistry on an Applied Biosystems model 3730 automated capillary DNA sequencer. All cDNA clones can be found at the MRC-PPU R&S website http://mrcppureagents.dundee.ac.uk/.

### Pharmacological inhibition

Small molecule inhibitors and compounds used are listed in the table S2. MG132 and cycloheximide treatments were at a final concentration of 10 μM and 350 μM, respectively.

### CRISPR-Cas9 genome editing

Genome editing was conducted using CRISPR (Clustered Regularly Interspaced Short Palindromic Repeat)-Cas9 system (41). *Rlim^-/y^*, RNF12 wild-type knock-in (WT-KI) and R575C-KI (10), RNF12 W576Y-KI and *Rlim^-/y^;Zfp42^-/-^* double knock-out (17) mESCs were described previously using guide RNA sequences summarized in the table S2. For knock-in experiments a third vector containing the donor DNA sequence followed by an IRES-EGFP cassette was co-expressed (table S2). CRISPR-Cas9 plasmid DNAs were transfected into mESCs using Lipofectamine (LTX) according to the manufacturer’s instructions and cultured for 24 h. Transfected mESCs were selected with 3 μg/ml puromycin for 48 h and then subjected into single-cell sorting using a FACS instrument into gelatinized 96-well cell culture plates. Candidate clones were analyzed by immunoblotting and genomic DNA sequencing to confirm gene editing. Sequencing was carried out by MRC-PPU DNA sequencing service. Endogenous N-terminal HA-tagged RNF12 wild-type knock-in (HA-RNF12 WT-KI) mESCs were generated from RNF12 wild-type knock-in (RNF12 WT-KI) (17) using ssODN CRISPR. 1 μg of DU 69536 plasmid (guide RNA sequence: GAGAATCTGAGTTCTCCATCT) and 1 μl of 100 μM HA-RNF12 WT-KI ssODN donor (TAAAGGTACGCAATGCATAATGGCGTCTGTTTTCAATTTGGTCTTTTTGCTTTTTAGATAATTTT CCACTTGATCACCAAGATGGtcTACCCATACGACGTcCCAGATTACGCTGAGAACTCAGATTCT AACGATAAAGGAAGTGACCAGTCTGCAGCTCAGCGCAGAAGTCAAATGGATCGCTTGGATCG GGAAGAGGC) were transfected into mESCs using Lipofectamine (LTX).

### Immunofluorescence

For mESCs cultured in LIF-FCS, 2x10^5^ cells/cm^2^ were plated into 12-well plates containing a single gelatin-coated coverslip per well. For transfected mESCs, this procedure was conducted 24 h post-transfection. Cells were left to attach for 24 h, media removed, and cells washed twice with PBS. Cells were then fixed to coverslips using 4% (w/v) paraformaldehyde (PFA), diluted in PBS and incubated for 20 mins at room temperature in the dark. For PGCLCs, cell clusters were dissociated using Trypsin-EDTA (Gibco) and washed twice with PBS. Cells were then fixed to coverslips in suspension using 4% (w/v) paraformaldehyde (PFA), diluted in PBS and incubated for 20 mins at room temperature in the dark. After 15 mins fixation cells were transferred to Poly-l-lysine (Sigma) treated coverslips and centrifuged for the remaining 5 mins into the coverslip.

Fixed cells were then washed three times with PBS and permeabilized in 0.5% Triton X-100 (w/v) diluted in PBS for 5 mins at room temperature. Permeabilized cells were blocked using 1% (w/v) fish gelatin diluted in PBS for at least 30 mins at room temperature inside a humid chamber. Cells were then stained with primary antibody (table S2) in 1% (w/v) fish gelatin diluted in PBS for 2 h at room temperature and in a humid chamber. After washing with PBS three times, cells were stained with secondary antibody tagged to fluorophore (table S2) diluted in 1% fish gelatin for 1 h at room temperature in a humid chamber and in darkness. DNA was stained using 0.1 μg/ml Hoechst diluted in PBS for 5 mins at room temperature in the dark. Coverslips were mounted on microscope slides using microscopy grade mounting media and left to dry at room temperature in the dark for 24 h before imaging. Images were acquired in a Zeiss 710 confocal microscope using Zen software (Zeiss).

### Immunohistochemistry

USP26 protein expression was analysed by DAB immunohistochemistry of mouse testes sections prepared from two biological replicates of 8 week old *Rlim*^fl/y^ and *Rlim*^-/^ littermates (15) using USP26 antibody (table S2; 1:100 dilution). These experiments were performed as described previously (9).

### Protein extraction

mESCs were harvested using lysis buffer (20 mM Tris-HCl (pH 7.4), 150 mM NaCl, 1 mM EDTA, 1% (v/v) NP-40, 0.5% (w/v) sodium deoxycholate, 10 mM β-glycerophosphate, 10 mM sodium pyrophosphate, 1 mM NaF, 2 mM Na3VO4, and 0.1 U/ml Complete Protease Inhibitor Cocktail Tablets (Roche)). Mouse organs were harvested from 19-week-old C57BL/6J mice and snap frozen in liquid nitrogen, resuspended in lysis buffer and lysed using a Polytrone PT 1200 E homogeniser (Kinematica AG). Mouse studies were previously approved by the University of Dundee ethical review committee, and further subjected to approved study plans by the Named Veterinary Surgeon and Compliance Officer (Dr. Ngaire Dennison) and performed under a UK Home Office project license in accordance with the Animal Scientific Procedures Act (ASPA, 1986). Protein concentration of protein extracts was determined using BCA Protein Assay Kit (Pierce) according to manufacturer’s directions. Protein concentration was calculated using a standard BSA protein curve.

### Immunoprecipitation

For RNF12 immunoprecipitations, 20 μl protein G agarose beads (MRC-PPU R&S) were washed three times in lysis buffer and incubated with 1 mg of mESC lysate and 2 μg of RNF12 antibody (table S2) overnight at 4 °C. For FLAG- or HA- tag pulldowns, 10 μl of pre-coupled FLAG-M2 agarose, 20 μl HA-Sepharose beads (MRC-PPU R&S) or 10 μl Pierce Anti-HA magnetic beads or IgG coupled to protein G magnetic beads (Thermo Scientific) were washed three times with lysis buffer and incubated with 1 mg of mESC lysate overnight at 4°C. Modified haloalkane dehalogenase (HALO)-tagged tandem ubiquitin binding element (HALO-TUBE) beads and HALO-MUD1 beads were produced as described (42). 1 mg of mESC lysate was mixed with 40 μl of HALO-TUBE or HALO-MUD1 beads and incubated overnight at 4 °C on a rotating wheel. In all cases, beads were washed three times with lysis buffer containing 500 mM NaCl. At each step, beads were centrifuged at 2000 rpm for 2 mins or separated using a magnetic stand and supernatant was discarded. Finally, proteins bound to the beads were eluted by the addition of LDS sample buffer and boiling the mixture 5 mins at 95 °C.

### Size Exclusion Chromatography

Size exclusion chromatography (SEC) running buffer (50 mM Tris-HCl (pH 7.5), 10% Glycerol (v/v), 150 mM NaCl, 1 mM DTT was freshly prepared and degassed by passing through a 0.45 μm PVDF filter (Millipore). 24 h prior to use, an ÄKTA™ pure protein purification instrument was equilibrated using SEC running buffer. A single confluent 15 cm plate of mESCs was harvested in SEC cell collection buffer (1 mM EGTA, 1 mM EDTA in PBS). Detached cells were collected and centrifuged 1000 rpm for 5 mins at 4 °C. Supernatant was discarded and pellet resuspended in 5 volumes of lysis buffer (20 mM Tris-HCl (pH 7.4), 150 mM NaCl, 1 mM EDTA, 1% NP-40 (v/v), 0.5% sodium deoxycholate (w/v), 10 mM β-glycerophosphate, 10 mM sodium pyrophosphate, 1 mM NaF, 2 mM Na3VO4 and 0.1 U/ml Complete Protease Inhibitor Cocktail Tablets (Roche)) and incubated for 15 mins on ice. After lysis, mixture was subjected to centrifugation at 14,000 rpm for 15 mins at 4 °C. Supernatant was then collected and passed through a 0.45 μm filter. Finally, clarified sample was mixed with Gel Filtration Standards (molecular weight range 670 – 1.35 kDa #1511901, Bio-Rad) and injected into an ÄKTA™ pure protein purification instrument loaded with a Superose 6 column through which a standard size exclusion chromatography protocol was run and 30 fractions collected per sample.

### Chromatin fractionation

For fractionation of soluble and chromatin-associated proteins, mESCs were lysed in CSK buffer (0.5% Triton X-100, 10 mM Hepes (pH 7.4), 100 mM NaCl, 300 mM sucrose, 3 mM MgCl2, 1 mM EGTA, 5 mM NaF, 1 mM Na3VO4, 1mM sodium pyrophosphate, 1 mM β-glycerophosphate plus 0.1 U/ml Complete Protease Inhibitor Cocktail Tablets (Roche)), and incubated on ice for 5 minutes. Samples were centrifuged at 13000 rpm for 5 mins and supernantant (soluble fraction) saved. The pellet was washed 3 times with CSK buffer and resuspended in NaCl buffer (0.1% Triton X-100, 50mM TRIS (pH 7.4), 250 mM NaCl, 1 mM EDTA, 5 mM NaF, 1 mM Na3VO4, 1 mM sodium pyrophosphate, 1 mM β-glycerophosphate plus 0.1 U/ml Complete Protease Inhibitor Cocktail Tablets (Roche)) with Benzonase (Sigma E1014-5KU) 1:500. Samples were incubated on ice for 30 mins and resuspended every 10 mins. Samples were then centrifuged at 13000 rpm for 15 mins and the supernatant (chromatin fraction) was saved. Soluble fraction was centrifuged again before analysis to remove any chromatin contamination.

### Immunoblotting

NuPAGE™ 4-12% SDS-PAGE gels were transferred onto PVDF membrane and incubated with primary antibody (table S2) diluted in 5% (w/v) non-fat milk buffer in TBS-T overnight at 4 °C. After incubation, membranes were washed 3 times with TBS-T buffer (20 mM Tris-HCl (pH 7.5), 150 mM NaCl supplemented with 0.2% (v/v) Tween-20 (Sigma Aldrich)) and incubated for 1 h with secondary horseradish Peroxidase (HRP)-conjugated antibodies (table S2) at room temperature. Finally, membranes were washed 3 times with TBS-T and subjected to chemiluminescence detection with Immobilon Western Chemiluminescent HRP (Horseradish Peroxidase) Substrate (Merck Millipore) using a Gel-Doc XR+ System (BioRad). Acquired images were analyzed and quantified using ImageLab software (BioRad).

### RNF12 in vitro autoubiquitylation assays

Two versions of the mRNF12 auto-ubiquitylation assay were performed. 140 nM RNF12 was incubated with 500 nM GST-mUSP26, GST-hUSP2 or GST for 1 h at 4°C. The reaction was started by adding a mix containing 0.1 μM UBE1, 0.05 μM UBE2D1, 2 μM FLAG-ubiquitin, 0.5 mM TCEP (pH 7.5), 5 mM ATP (both from Sigma Aldrich), 50 mM Tris-HCl (pH 7.5) and 5 mM MgCl2 was then added and incubated for 1 h at 30°C. Either RNF12 or ATP were omitted in control samples. Reactions were stopped by adding SDS sample buffer and boiled for 5 min. This version is labelled “BEFORE” in Fig. 4A. Alternatively, 140 nM RNF12, 0.1 μM UBE1, 0.05 μM UBE2D1, 2 μM FLAG-ubiquitin, 0.5 mM TCEP (pH 7.5), 5 mM ATP (both from Sigma Aldrich), 50 mM Tris-HCl (pH 7.5) and 5 mM MgCl2 was mixed and incubated for 1 h at 30°C. Either RNF12 or ATP were omitted in control samples. After incubation, ATP was depleted with 4.5 U/ml apyrase (New England Biolabs) for 10 min at room temperature. Finally, 500 nM GST-mUSP26, GST-hUSP2 or GST were added and the reaction incubated for a further 1 h at 4°C. Reactions were stopped by adding SDS sample buffer and boiled for 5 min. This version is labelled “AFTER” in Fig. 4A Samples were loaded on 4%–12% Bis-Tris gradient gels (Thermo Fisher Scientific) and analyzed by immunoblotting.

### RNA extraction and qRT-PCR

mESCs were seeded in 6-well plates and left to grow 24-48 h until confluent. RNA was extracted using E.Z.N.A.® MicroElute Total RNA Kit (Omega Bio-Tek) according to manufacturer’s instructions. RNA samples were subjected to reverse transcription using iScript™ cDNA Synthesis Kit (BioRad) according to the manufacturer’s instructions. qPCR primer sequences were identified from the PrimerBank database (https://pga.mgh.harvard.edu/primerbank) or designed using Primer3 software with a melting temperature between 58-62 °C. For each primer, the integrity and specificity were confirmed in silico by NCBI Primer-Blast software (https://www.ncbi.nlm.nih.gov/tools/primer-blast). All primers are 20-24 bases long with an overlap of seven bases at the intron/exon boundary producing an amplicon of 100-300 bases. Primers were supplied by Invitrogen or Thermo Scientific according to availability. qPCR primer sequences are listed in table S2. qPCR reactions were carried out using SsoFast EvaGreen Supermix (BioRad) or TB Green Premix Ex Taq (Takara). In either case, each sample consisted of a 10 μl reaction containing 1 μl cDNA with 400 nM forward and reverse primers, 5 μl SYBR Green and nuclease free water. Each sample was prepared in technical duplicate. qPCR and absorbance detection were carried out in a CFX384 real-time PCR system (BioRad). The ΔΔCt method, also known as the Pfaffl method (43) was used to calculate relative RNA abundance with *Gapdh* expression as a loading control. Data were analyzed in Excel software and plotted in GraphPad Prism v8.00 software.

### Protein preparation for quantitative proteomic profiling

mESCs were washed in PBS and then lysed in 8.5 M urea, 50 mM ammonium bicarbonate (pH 8.0) supplemented with protease inhibitors. Lysate was sonicated using Biosonicator operated at 50% power for 30 s on/off each on ice water bath for 5 min. The lysates were then centrifuged at 14,000 rpm for 10 mins at 4 °C and supernatants collected. Protein concentration of the lysate was determined by BCA protein assay. Proteins were reduced with 5 mM DTT at 55 °C for 30 min and cooled to room temperature. Reduced lysates were then alkylated with 10 mM iodoacetamide at room temperature for 30 mins in the dark. The alkylation reaction was quenched by the addition of another 5 mM DTT. After 20 min of incubation at room temperature, the lysate was digested using Lys-C with the weight ratio of 1:200 (Enzyme:lysate) at 37 °C for 4 h. The samples were further diluted to 1.5 M Urea with 50 mM ammonium bicarbonate (pH 8.0), and the sequencing-grade trypsin was added with the weight ratio of 1:50 (enzyme:lysate) and incubated overnight at 37 °C. The digest was acidified to pH 3.0 by addition of TFA to 0.2% and gently mix at room temperature for 15 min; the resulting precipitates were removed by centrifugation at 7100 RCF for 15 min. The acidified lysate was then desalted using a C18 SPE cartridge (Waters) and the eluate was aliquoted into 100 μg and dried by vacuum centrifugation. To check the digests, 1 μg of each sample was analyzed by mass spectrometry prior to TMT labelling.

100 μg of peptide from each sample was re-suspended into 100 mM Triethylammonium bicarbonate buffer (pH 8.5). Then 0.8 mg of TMT tag (Thermo) dissolved in 41 μl of anhydrous acetonitrile was transferred to the peptide sample and incubated with 60 min at room temperature. The TMT labelling reaction was quenched with 5% hydroxylamine. 1 μg of each labelled sample was analyzed by mass spectrometry to assess the labelling efficiency before pooling. After checking the labelling efficiency, the TMT-labelled peptides were mixed together and dried by vacuum centrifugation. After dryness, the mixture of TMT-labelled peptides was dissolved into 0.2% TFA and then desalted using a C18 SPE cartridge. The desalted peptides were subjected to orthogonal basic pH reverse phase fractionation, collected in 96-well plate and consolidated for a total of 20 fractions for vacuum dryness.

### LC-MS/MS, data processing, and analysis

Each fraction was dissolved in 0.1% FA and quantified by Nanodrop. 1 μg of peptide was loaded on C18 trap column at a flow rate of 5 μl/min. Peptide separations were performed over EASY-Spray column (C18, 2 μm, 75 mm × 50 cm) with an integrated nano electrospray emitter at a flow rate of 300 nl/min. The LC separations were performed with a Thermo Dionex Ultimate 3000 RSLC Nano liquid chromatography instrument. Peptides were separated with a 180 min segmented gradient as follows: 7%~25% buffer B (80% ACN/0.1% FA) in 125 min, 25%~35% buffer B for 30 min, 35%~99% buffer B for 5 min, followed by a 5 min 99% wash and 15 min equilibration with buffer A (0.1% FA).

Data acquisition on the Orbitrap Fusion Tribrid platform with instrument control software version 3.0 was carried out using a data-dependent method with multinotch synchronous precursor selection MS3 scanning for TMT-9plex tags. The mass spectrometer was operated in data-dependent most intense precursors Top Speed mode with 3 s per cycle. The survey scan was acquired from m/z 375 to 1500 with a resolution of 120,000 resolving power with AGC target 400,000. The maximum injection time for full scan was set to 60 ms. For the MS/MS analysis, monoisotopic precursor selection was set to peptide. AGC target was set to 50,000 with the maximum injection time 120 msec. Charge states unknown and 1 or higher than 7 were excluded. The MS/MS analyses were performed by 1.2 m/z isolation with the quadrupole, normalized HCD collision energy of 37% and analysis of fragment ions in the Orbitrap using 15,000 resolving power with auto normal range scan starting from m/z 110. Dynamic exclusion was set to 60 s. For the MS3 scan, the MS3 precursor population from MS2 scan ranging from m/z 300-100 was isolated using the SPS waveform and then fragmented by HCD. The HCD normalized collision energy was set to 65. The MS3 scan were acquired from m/z 100 to 500 with a resolution of 50,000 and AGC target 50,000. The maximum injection time for full scan was set to 86 ms.

Data from the Orbitrap Fusion were processed using Proteome Discoverer Software (version 2.2). MS2 spectra were searched using Mascot against a UniProt Mouse database appended to a list of common contaminants (10,090 total sequences). The searching parameters were specified as trypsin enzyme, two missed cleavages allowed, minimum peptide length of 6, precursor mass tolerance of 20 ppm, and a fragment mass tolerance of 0.05 Daltons. Oxidation of methionine and TMT at lysine and peptide N-termini were set as variable modifications. Carbamidomethylation of cysteine was set as a fixed modification. Peptide spectral match error rates were determined using the target-decoy strategy coupled to Percolator modeling of positive and false matches. Data were filtered at the peptide spectral match-level to control for false discoveries using a q-value cut off of 0.01, as determined by Percolator. For quantification, the signal-to-noise values higher than 10 for unique and razor peptides were summed within each TMT channel, and each channel was normalized with total peptide amount. Quantitation was further performed by adjusting the calculated p-values according to Benjamini-Hochberg. The significance regulated proteins with p-value less than 0.05 were further manually investigated with the standard deviations of biological replicates.

For comparison of this RNF12 quantitative proteomic dataset with that of Gontan et al., 2018 (21), proteins with >1.5 fold increase and t-test significance p-value <0.05 in RNF12 expressing Rlim-/y mESCs compared to control Rlim^-/y^ mESCs (this dataset) were intersected with proteins with >1.5 fold increase in *Rlim^+/+^* compared to *Rlim^-/-^* mESCs (21). Significance p-value was not considered as a threshold for data from (21) to include the positive control REX1. Data was intersected using Venny https://bioinfogp.cnb.csic.es/tools/venny/index.html.

### RNA-sequencing (RNA-SEQ) and gene ontology (GO-term) analysis

RNA-SEQ data for *Rlim^-/y^* mESCs expressing either empty vector or RNF12 WT was described previously (17) (see Gene Expression Omnibus (GEO) accession GSE149554). Reads were aligned using Spliced Transcripts Alignment to a Reference (STAR) software. Differential gene expression was estimated using DESeq2 package and further statistical analysis and plot generation were performed with The SARTools R package. Gene Ontology (GO) analysis were carried out using the GO stat R package.

### Chromatin immunoprecipitation DNA sequencing (ChlP-SEQ) analysis

Chip-Seq data analyzed were from (12), GEO accession number GSM892958. The quality of sequencing was analyzed using FastQC software. The sequences were aligned to the mm10 reference genome using Bowtie 2 with standard settings (44). PCR duplicates were removed with Picard (http://broadinstitute.github.io/picardZ). The reads from each group of samples were merged to call peaks using MACS2 software (45). Peak finding was performed applying broad peak calling parameters. For basic annotation of identified peaks, the HOMER function annotatePeaks.pl was used (46).

### Data analysis

Data is presented as mean ± S.E.M. with individual points representing a single biological replicate. In qPCR experiments two technical replicates were run per sample and averaged. Immunofluorescence images were processed using ImageJ (ImageJ) and Photoshop CS5.1 (Adobe) software. The percentage of cells expressing a certain protein was calculated as the ratio between cells positive for protein expression (containing antibody fluorescence) and the total number of cells (containing DNA fluorescence provided by Hoechst stain). Parameters were quantified using Fiji (ImageJ) software. Graphs were created using Prism software (GraphPad). In all cases, statistical significance was determined through ANOVA followed by Tukey’s post hoc test or student’s T-test using Prism software (GraphPad) and significant differences were considered when p<0.05.

## Supplementary Material

Data File S1

Supplementary Material

## Data Availability

The mass-spectrometry data have been deposited in EBI-PRIDE (https://www.ebi.ac.uk/pride/) under accession code PXD032317. RNA sequencing data analyzed in this paper were published previously (17) and deposited in GEO (https://www.ncbi.nlm.nih.gov/geo/) under accession code GSE149554. All other data needed to evaluate the conclusions in the paper are present in the paper or the Supplementary Materials.
