# Supplementary material for: An RNF12-USP26 amplification loop drives germ cell specification and is disrupted by disease-associated mutations
Source: Sci Signal. Author manuscript; Available in PMC 2022 Oct 5. (PMC7613676; doi:10.1126/scisignal.abm5995)
Supplement: Supplementary Material [file EMS155062-supplement-Supplementary_Material.pdf]

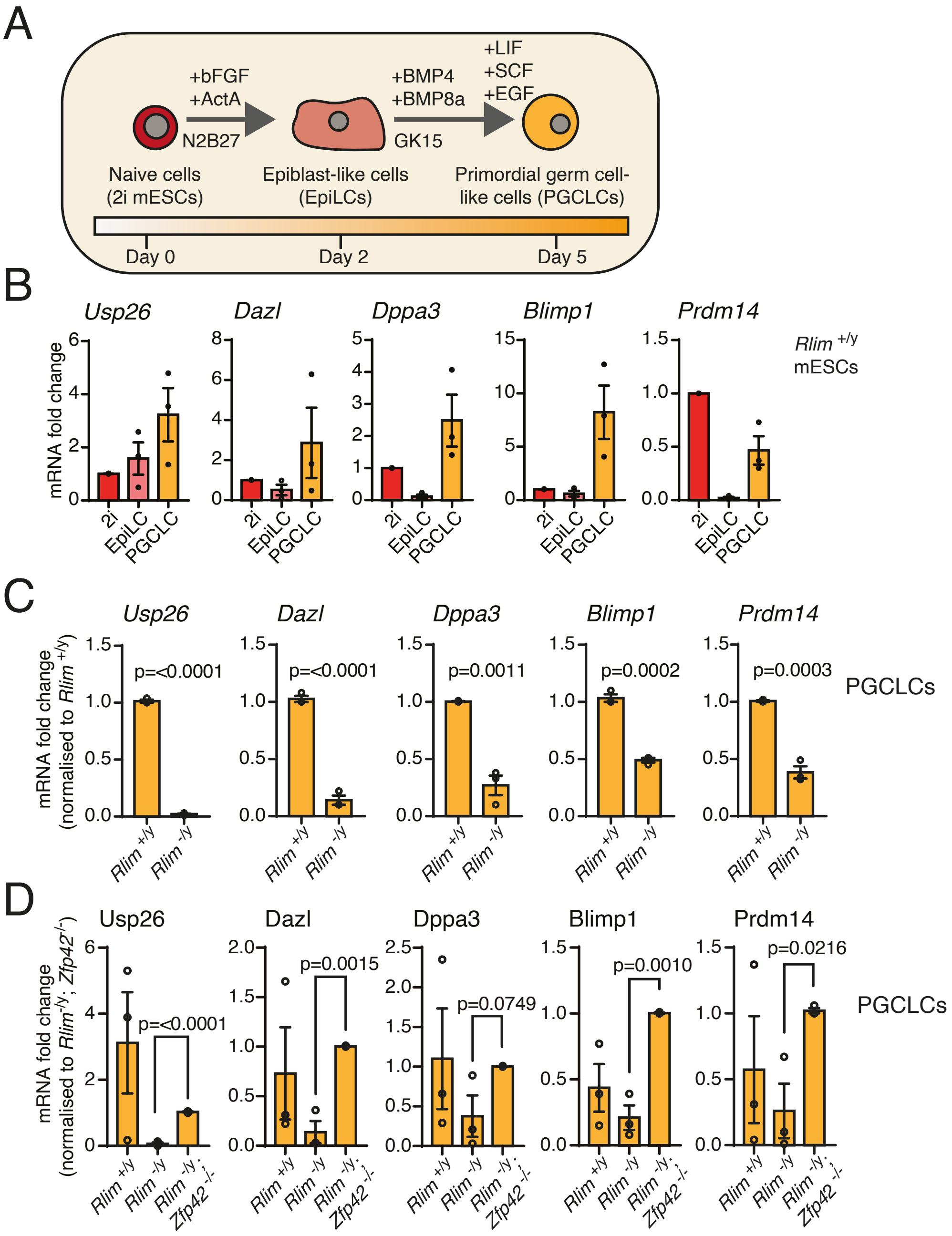
A
+bFGF
+ActA
N2B27
+BMP4
+BMP8a
GK15
+LIF
+SCF
+EGF
Naive cells (2i mESCs)
Epiblast-like cells (EpiLCs)
Primordial germ cell-like cells (PGCLCs)
Day 0
Day 2
Day 5
B
Usp26
Dazl
Dppa3
Blimp1
Prdm14
mRNA fold change
2i
EpiLC
PGCLC
Rlim +/y mESCs
C
Usp26
Dazl
Dppa3
Blimp1
Prdm14
mRNA fold change (normalised to Rlim +/y)
p=<0.0001
p=<0.0001
p=0.0011
p=0.0002
p=0.0003
Rlim +/y
Rlim -/y
PGCLCs
D
Usp26
Dazl
Dppa3
Blimp1
Prdm14
mRNA fold change (normalised to Rlim -/y; Zfp42-/-)
p=<0.0001
p=0.0015
p=0.0749
p=0.0010
p=0.0216
Rlim +/y
Rlim -/y
Rlim -/y; Zfp42 -/-
PGCLCs

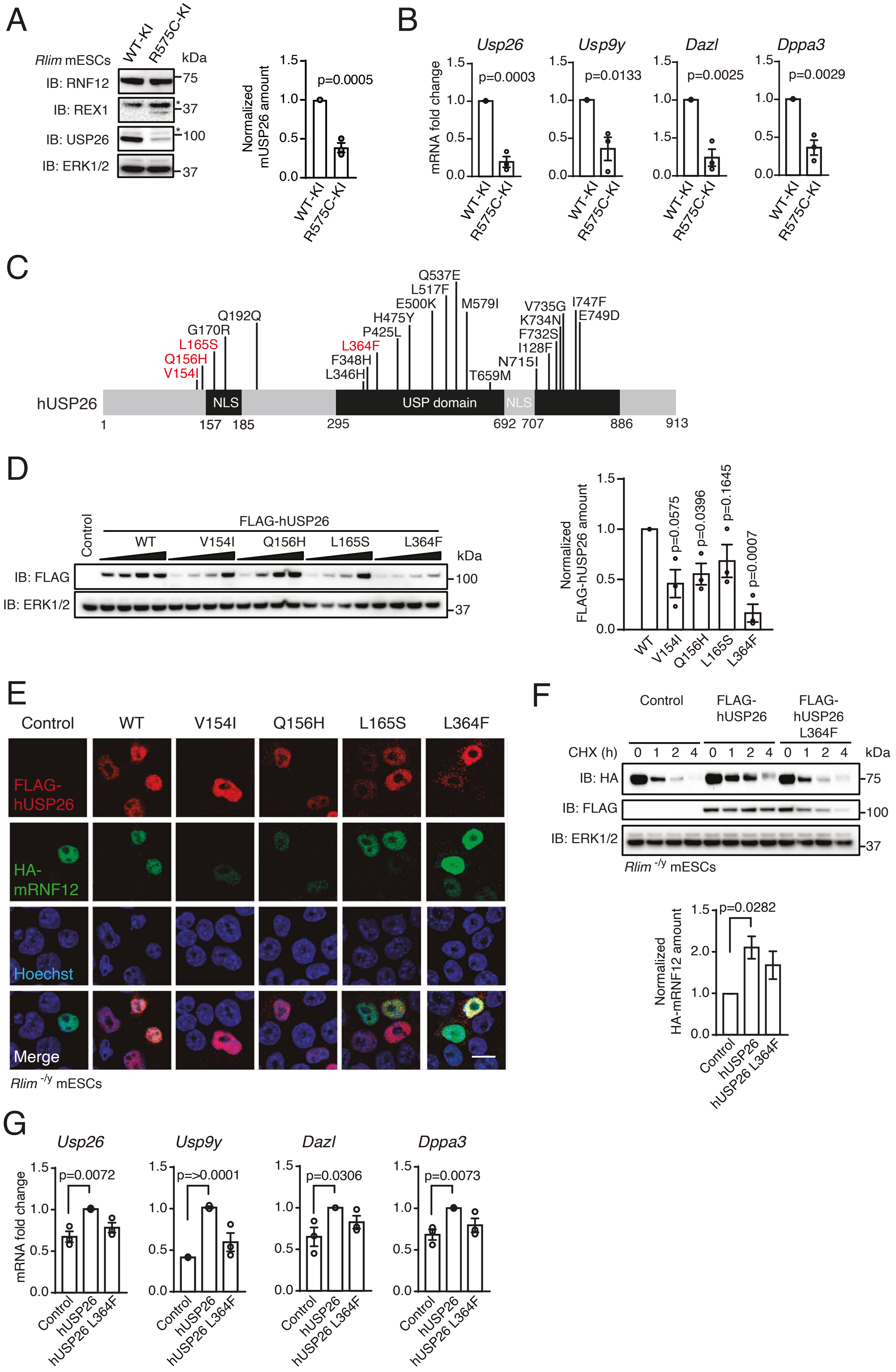

A
Rlim mESCs
WT-KI
R575C-KI
kDa
IB: RNF12
IB: REX1
IB: USP26
IB: ERK1/2
75
37
100
37
Normalized mUSP26 amount
p=0.0005
B
Usp26
Usp9y
Dazl
Dppa3
mRNA fold change
p=0.0003
p=0.0133
p=0.0025
p=0.0029
C
hUSP26
NLS
USP domain
NLS
V154I
Q156H
L165S
G170R
Q192Q
L346H
F348H
L364F
P425L
H475Y
E500K
L517F
Q537E
M579I
T659M
N715I
I128F
F732S
K734N
V735G
I747F
E749D
1
157
185
295
692
707
886
913
D
Control
FLAG-hUSP26
WT
V154I
Q156H
L165S
L364F
kDa
IB: FLAG
IB: ERK1/2
100
37
Normalized FLAG-hUSP26 amount
p=0.0575
p=0.0396
p=0.1645
p=0.0007
E
Control
WT
V154I
Q156H
L165S
L364F
FLAG-hUSP26
HA-mRNF12
Hoechst
Merge
Rlim -/y mESCs
F
Control
FLAG-hUSP26
FLAG-hUSP26 L364F
CHX (h)
kDa
IB: HA
IB: FLAG
IB: ERK1/2
75
100
37
Rlim -/y mESCs
Normalized HA-mRNF12 amount
p=0.0282
Control
hUSP26
hUSP26 L364F
G
Usp26
Usp9y
Dazl
Dppa3
mRNA fold change
p=0.0072
p=>0.0001
p=0.0306
p=0.0073
Control
hUSP26
hUSP26 L364F

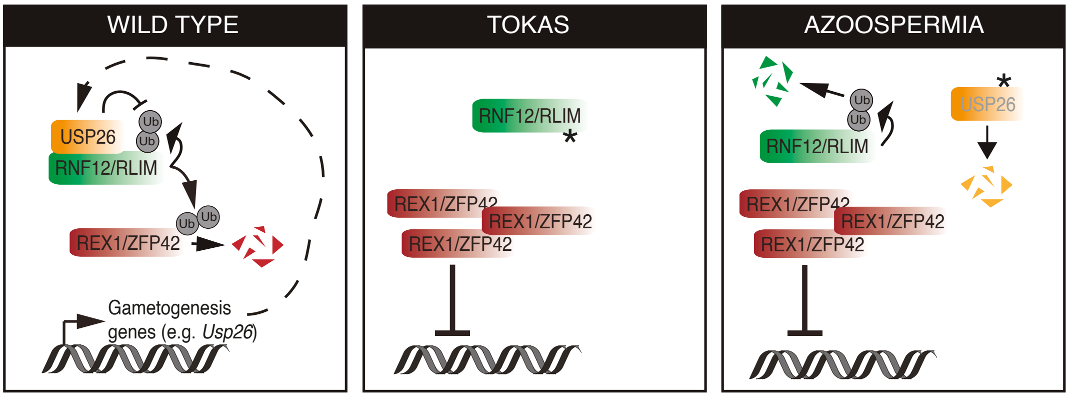

WILD TYPE
USP26
Ub
Ub
RNF12/RLIM
Ub
Ub
REX1/ZFP42
Gametogenesis
genes (e.g. *Usp26*)
TOKAS
RNF12/RLIM
*
REX1/ZFP42
REX1/ZFP42
REX1/ZFP42
AZOOSPERMIA
Ub
Ub
*
USP26
RNF12/RLIM
REX1/ZFP42
REX1/ZFP42
REX1/ZFP42

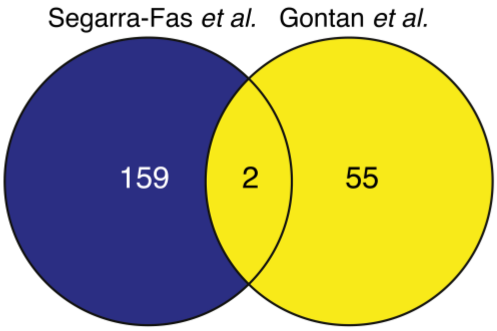

fig. S1. Comparison of RNF12 quantitative proteomic datasets. RNF12-induced proteins from this study were intersected with RNF12-induced proteins from Gontan *et al*., 2018 (*21)*.

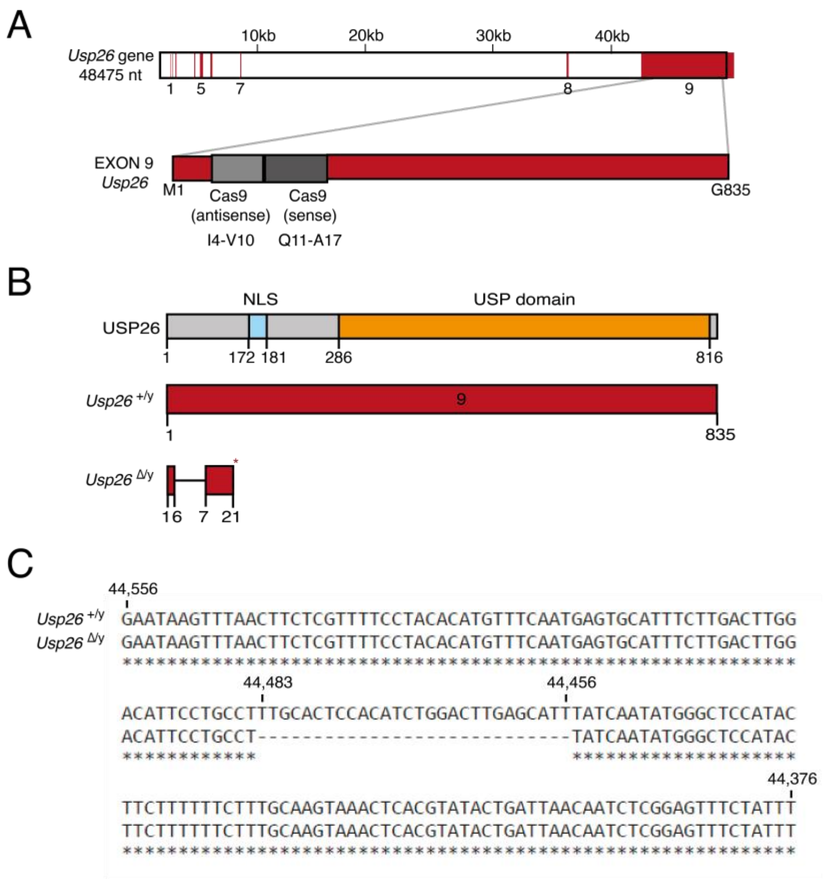

fig. S2. CRISPR-Cas9 generation of *Usp26*$^{\Delta/y}$ mESCs. (**A**) Schematic showing Exon 9 within the mouse *Usp26* gene and CRISPR guide RNA sequences. (**B**) USP26 domain organization and predicted protein products produced in *Usp26*$^{+/y}$ and *Usp26*$^{\Delta/y}$ mESCs. (**C**) Genomic DNA sequencing data for *Usp26*$^{+/y}$ and *Usp26*$^{\Delta/y}$ mESCs.

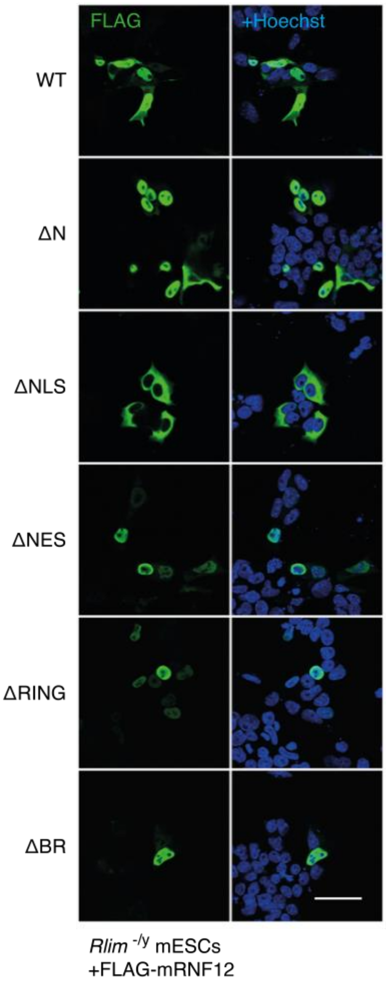

fig. S3. Subcellular localization of RNF12 deletion mutants. HA-immunofluorescence of *Rlim*$^{-/y}$ mESCs expressing HA-mRNF12 WT (1-600), Δ1-206 (ΔN), Δ206-226 (ΔNLS), Δ502-513 (ΔNES) Δ546-587 (ΔRING), or Δ326-423 (ΔBR). Nuclei are stained with Hoechst. Scale bar, 50 μm. Data are representative of n = 3 independent experiments.

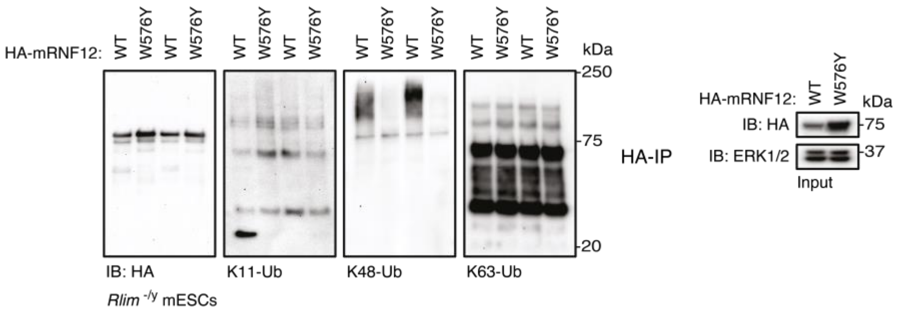

fig. S4. RNF12 is modified by K48-linked ubiquitin. Immunoblotting HA immunoprecipitates from *Rlim*$^{-/y}$ mESCs expressing the indicated HA-mRNF12 constructs for HA and K11-, K48-, and K63-linked ubiquitin. Data are representative of n = 2 independent experiments.

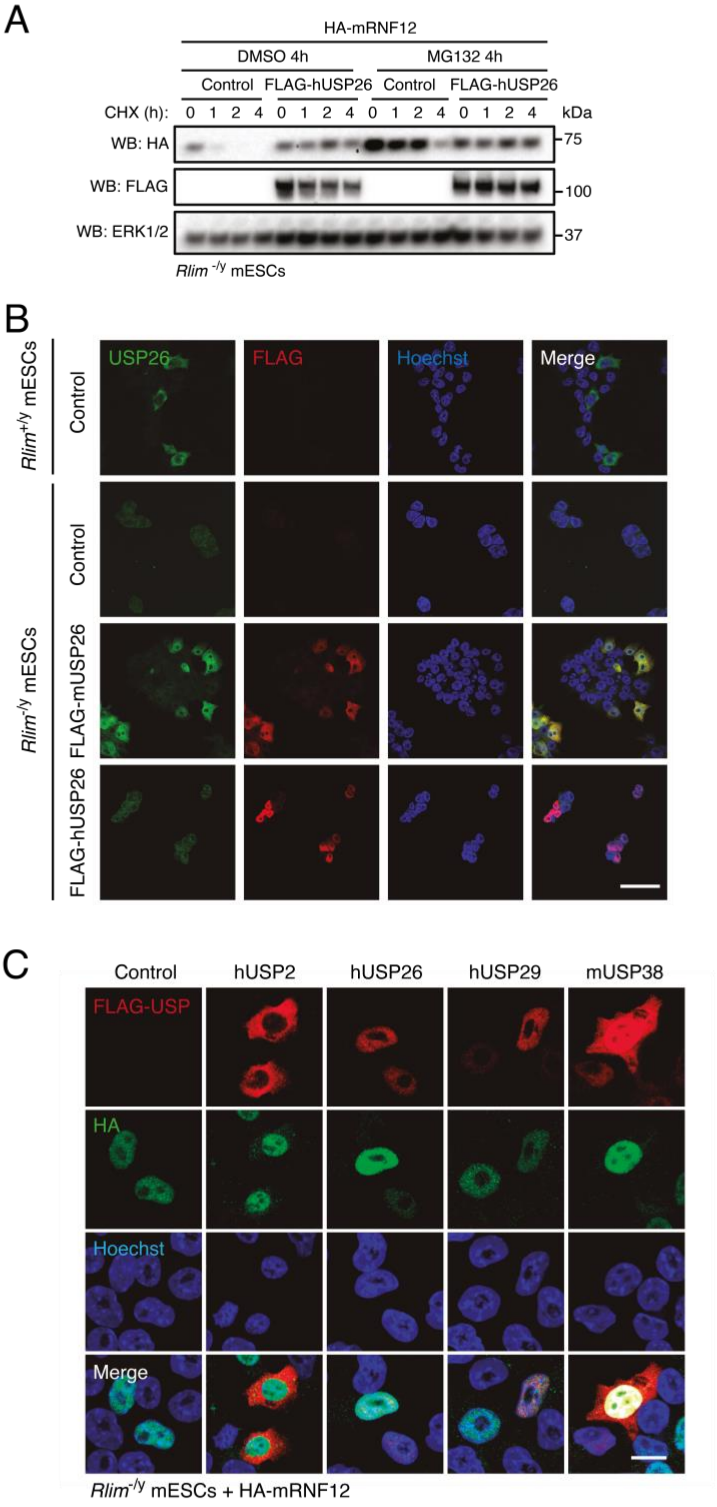
A
HA-mRNF12
DMSO 4h
MG132 4h
Control
FLAG-hUSP26
Control
FLAG-hUSP26
CHX (h): 0 1 2 4 0 1 2 4 0 1 2 4 0 1 2 4
kDa
WB: HA
75
WB: FLAG
100
WB: ERK1/2
37
Rlim -/y mESCs
B
USP26
FLAG
Hoechst
Merge
Rlim+/y mESCs
Control
Rlim-/y mESCs
Control
FLAG-mUSP26
FLAG-hUSP26
C
Control
hUSP2
hUSP26
hUSP29
mUSP38
FLAG-USP
HA
Hoechst
Merge
Rlim-/y mESCs + HA-mRNF12

fig. S5. Function and localization of USP26 and other deubiquitylases. (**A**) *Rlim*$^{-/y}$ mESCs expressing empty vector (Control) or FLAG-hUSP26 and HA-mRNF12 WT were treated with DMSO or MG132 for 4h and cycloheximide (CHX) for the indicated times. HA-mRNF12, FLAG-hUSP26, and ERK1/2 were analyzed by immunoblotting. Data are representative of n = 3 independent experiments. (**B**) Immunofluorescence of *Rlim*$^{+/y}$ mESCs and *Rlim*$^{-/y}$ mESCs expressing empty vector (Control), FLAG-hUSP26, or FLAG-mUSP26. FLAG-hUSP26 and FLAG-mUSP26 were detected by FLAG (red); FLAG-mUSP26 and endogenous mUSP26 were detected by total USP26 (green) immunofluorescence. Nuclei are stained with Hoechst. Scale bar, 50 μm. Data are representative of n = 3 independent experiments. (**C**) Immunofluorescence of *Rlim*$^{-/y}$ mESCs expressing HA-mRNF12 and either empty vector (Control), FLAG-hUSP2, FLAG-hUSP26, FLAG-hUSP29, or FLAG-mUSP38. FLAG-USPs were detected by FLAG; HA-mRNF12 was detected by HA. Nuclei are stained with Hoechst. Scale bar, 10 μm. Data are representative of n = 3 independent experiments.

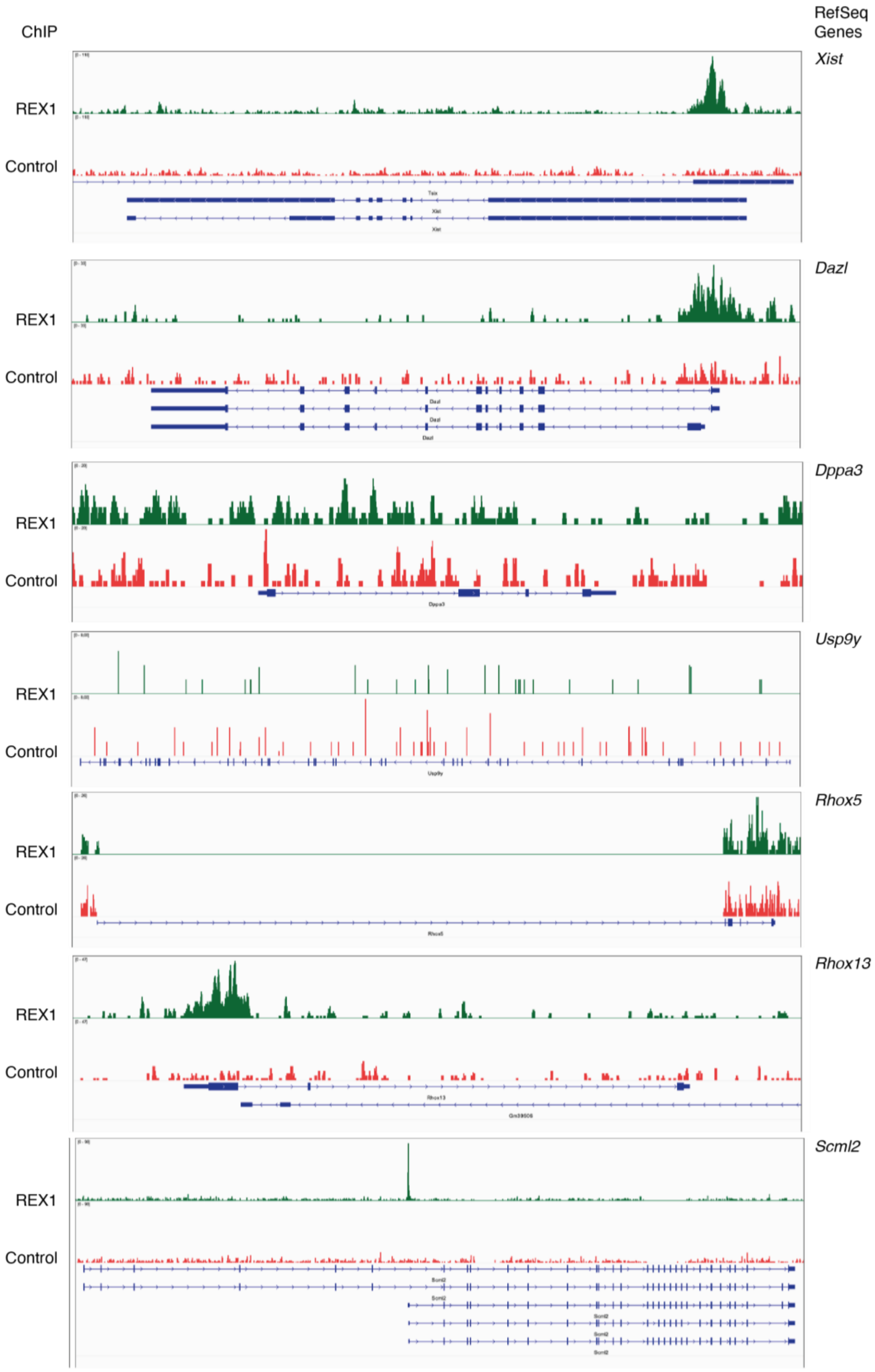

fig. S6. REX1 associates with gametogenesis gene promoters. REX1 and control chromatin immunoprecipitation and DNA sequencing, showing assigned peaks for the indicated genes.

Table S1. Summary of RNF12-dependent gametogenesis genes.

Gene	Protein	Uniprot ID	Function	Reference
Kdm5d	Lysine-specific demethylase 5D	Q62240	H3K4 demethylase, may play a role in spermatogenesis	https://pubmed.ncbi.nlm.nih.gov/3951555/
Usp9y	Ubiquitin-specific peptidase 9, Y chromosome	F8VPU6	Gene found in the azoospermia factor (AZF) region on the Y chromosome, associated with Sertoli cell-only syndrome (SCO) and male infertility	https://pubmed.ncbi.nlm.nih.gov/11420393/
Tdrd12	Tudor domain-containing protein 1	Q9CWU0	Essential for germ cell development and maintenance in zebrafish	https://pubmed.ncbi.nlm.nih.gov/28590408/
Dppa3	Developmental pluripotency-associated protein 3	Q8QZY3	Primordial germ cell (PGC)-specific protein	https://pubmed.ncbi.nlm.nih.gov/12124616/
Dazl	Deleted in azoospermia-like	Q64368	RNA-binding protein, which is essential for gametogenesis in both males and females. Plays a central role during spermatogenesis	https://pubmed.ncbi.nlm.nih.gov/9288969/
Rhox5	Homoeobox protein Rhox5	P52651	Transcription factor required for ESC differentiation into primordial germ cells	https://pubmed.ncbi.nlm.nih.gov/24074865/
Rhox13	Homeobox protein Rhox13	F6YCR7	Transcription factor required for male germ cell differentiation	https://pubmed.ncbi.nlm.nih.gov/27486269/
Pramel3	PRAME-like 3	A2AHA7	Present in a 1.1Mb region of the X chromosome	https://pubmed.ncbi.nlm.nih.gov/23677977/

			required for male meiosis	
Prame	Melanoma antigen preferentially expressed in tumors	Q9D4Z5	Deletion in mouse gene causes germ cell reduction in spermatogenesis	https://pubmed.ncbi.nlm.nih.gov/32017313/
Scml2	Transcriptional repressor Scml2	Q99MW4	Establishes the male germline epigenome through regulation of histone H2A ubiquitination	https://pubmed.ncbi.nlm.nih.gov/25703348/
4930550L24Rik	Mage-k1	Q99PA7	Expressed in the male reproductive system	https://bgee.org/?page=gene&gene_id=ENSMUSG00000046180
4930502E18Rik	Cancer/testis antigen 55	Q9D585	unknown function	
C030039L03Rik	KRAB domain-containing protein	Q3URA4	unknown function	
1700080O16Rik	1700080O16Rik protein	Q9D9G4	Expressed in the male reproductive system	https://bgee.org/?page=gene&gene_id=ENSMUSG00000031118
Usp26	Ubiquitin carboxyl-terminal hydrolase 26	Q99MX1	Mutation in mice leads to defective spermatogenesis	https://pubmed.ncbi.nlm.nih.gov/31551464/
Magea4	Melanoma antigen, family A, 4	F2Z493	Plays an important role in the initial phases of spermatogenesis	https://pubmed.ncbi.nlm.nih.gov/7627949/
Magea5	Melanoma-associated antigen 5	O89009	Regulates male germ cell apoptosis	https://pubmed.ncbi.nlm.nih.gov/27226137/
Magea8	MCG115467	O89012	Regulates male germ cell apoptosis	https://pubmed.ncbi.nlm.nih.gov/27226137/
Pramel7	Preferentially expressed antigen in melanoma-like protein 7	Q810Y8	Promotes maintenance and self-renewal of pluripotent embryonic stem cells (ESCs)	https://pubmed.ncbi.nlm.nih.gov/21425410/

Gm773	Gene model 773, (NCBI)	Q3TML4	Expressed in spermatids	https://bgee.org/?page=gene&gene_id=ENSMUSG00000073177
Xist	none		X-chromosome inactivation, regulated by RNF12-REX1 pathway	https://pubmed.ncbi.nlm.nih.gov/31737626/

Table S2. Key Resources Table. This table is a complete list of biological resources used in this study.

**Reagent type (species) or resource**	**Designation**	**Source or reference**	**Identifiers**	**Additional information**
gene (*Mus Musculus*)	*Xist*	GenBank	213742	
gene (*Mus Musculus*)	*1700013H16 Rik*	GenBank	75514	
gene (*Mus Musculus*)	*Usp9y*	GenBank	107868	
gene (*Mus Musculus*)	*Pramel3*	GenBank	83565	
gene (*Mus Musculus*)	*Magea4*	GenBank	17140	
gene (*Mus Musculus*)	*Pramel7*	GenBank	347712	
gene (*Mus Musculus*)	*Kdm5d*	GenBank	20592	
gene (*Mus Musculus*)	*Gm773*	GenBank	331416	
gene (*Mus Musculus*)	*Usp26*	GenBank	83563	
gene (*Mus Musculus*)	*Rhox13*	GenBank	73614	
gene (*Mus Musculus*)	*Rhox5*	GenBank	18617	
gene (*Mus Musculus*)	*Dazl*	GenBank	13164	
gene (*Mus Musculus*)	*Dppa3*	GenBank	73708	
gene (*Mus Musculus*)	*Scml2*	GenBank	107815	
gene (*Mus Musculus*)	*Tdrd12*	GenBank	71981	
gene (*Mus Musculus*)	*Usp29*	GenBank	57775	

gene (*Mus Musculus*)	*Nanog*	GenBank	71950	
gene (*Mus Musculus*)	*Klf4*	GenBank	16600	
gene (*Mus Musculus*)	*Fgf5*	GenBank	14176	
gene (*Mus Musculus*)	*Rlim*	GenBank	19820	
gene (*Mus Musculus*)	*Zfp42*	GenBank	22702	
gene (*Mus Musculus*)	*Gapdh*	GenBank	14433	
strain, strain background (*Escherichia coli*)	BL21(DE3)	Sigma-Aldrich	CMC0016	Electrocompetent cells
genetic reagent (*Mus Musculus,* male)	*Rlim fl/y*	PMID: 20962847		Male mouse from the laboratory of Ingolf Bach, UMMS, MA
genetic reagent (*Mus Musculus,* male)	*Rlim -/y*	PMID: 20962847		Male mouse from the laboratory of Ingolf Bach, UMMS, MA
cell line (Mus Musculus, male)	*Rlim +/y; Usp26 +/y;* WT mESC	Other	CCE line RRID:CVCL_C313	Parental Male Mouse Embryonic Stem Cell line from the laboratory of Janet Rossant, SickKids Research Institute, Toronto
cell line (Mus Musculus, male)	*Rlim -/y*	PMID: 29742418		Male Mouse Embryonic Stem Cell line from the Laboratory of Greg Findlay, MRC PPU, Dundee
cell line (Mus Musculus, male)	*Rlim -/y Zfp42-/-*	PMID: 33080171		Male Mouse Embryonic Stem Cell line from the Laboratory of Greg Findlay, MRC PPU, Dundee
cell line (Mus Musculus, male)	WT-KI; RNF12 WT-KI; *Rlim* mESC WT-KI	PMID: 29742418		Male Mouse Embryonic Stem Cell line from the Laboratory of Greg Findlay, MRC PPU, Dundee
cell line (Mus Musculus, male)	R575C-KI; RNF12 R575C-KI;	PMID: 29742418		Male Mouse Embryonic Stem Cell line from the Laboratory of Greg

	Rlim mESC R575C-KI			Findlay, MRC PPU, Dundee
cell line (Mus Musculus, male)	W576Y-KI; RNF12 W576Y-KI; *Rlim* mESC W576Y-KI	PMID: 33080171		Male Mouse Embryonic Stem Cell line from the Laboratory of Greg Findlay, MRC PPU, Dundee
cell line (Mus Musculus, male)	*Usp26 Δ/y*	This paper		Male Mouse Embryonic Stem Cell line from the Laboratory of Greg Findlay, MRC PPU, Dundee
cell line (Mus Musculus, male)	HA WT-KI; RNF12 WT-KI; *Rlim* mESC WT-KI	This paper		Male Mouse Embryonic Stem Cell line from the Laboratory of Greg Findlay, MRC PPU, Dundee
recombinant DNA reagent	Control; EV	MRC-PPU Reagents and Services	DU49023	pCAGGS puro
recombinant DNA reagent	HA-RNF12; HA-RNF12; WT	MRC-PPU Reagents and Services	DU50854	pCAGGS puro HA mouse Rnf12
recombinant DNA reagent	HA-RNF12 W576Y; HA-W576Y	MRC-PPU Reagents and Services	DU61086	pCAGGS puro HA mouse Rnf12 W576Y
recombinant DNA reagent	HA-RNF12 4xK-R; 4xK-R	MRC-PPU Reagents and Services	DU61130	pCAGGS puro HA mouse Rnf12 K71R K526R K544R K558R
recombinant DNA reagent	HA-RNF12 all K-R; all K-R	MRC-PPU Reagents and Services	DU61139	pCAGGS puro HA mouse Rnf12 K9R K71R K526R K544R K558R 561R
recombinant DNA reagent	HA-RNF12 ΔN	MRC-PPU Reagents and Services	DU53408	pCAGGS puro HA mouse Rnf12 A206-V600(end)
recombinant DNA reagent	HA-RNF12 ΔNLS	MRC-PPU Reagents and Services	DU53426	pCAGGS puro HA mouse Rnf12 delta A206-R226
recombinant DNA reagent	HA-RNF12 ΔNES	MRC-PPU Reagents and Services	DU53405	pCAGGS puro HA mouse Rnf12 delta L502-L513

recombinant DNA reagent	HA-RNF12 ΔRING	MRC-PPU Reagents and Services	DU53419	pCAGGS puro HA mouse Rnf12 M1-L543
recombinant DNA reagent	HA-RNF12 ΔBR	MRC-PPU Reagents and Services	DU53422	pCAGGS puro HA mouse Rnf12 delta Y326-A423
recombinant DNA reagent	HA-RNF12 4xSA	MRC-PPU Reagents and Services	DU58741	pCAGGS puro HA mouse Rnf12 S212/214/227/229A
recombinant DNA reagent	FLAG-RNF12; FLAG-RNF12; WT	MRC-PPU Reagents and Services	DU49070	pCAGGS puro 3XFLAG mouse Rnf12
recombinant DNA reagent	FLAG-RNF12 ΔN	MRC-PPU Reagents and Services	DU53409	pCAGGS puro 3FLAG mouse Rnf12 A206-V600(end)
recombinant DNA reagent	FLAG-RNF12 ΔNLS	MRC-PPU Reagents and Services	DU53416	pCAGGS puro 3XFLAG mouse Rnf12 delta A206-R226
recombinant DNA reagent	FLAG-RNF12 ΔNES	MRC-PPU Reagents and Services	DU53421	pCAGGS puro 3XFLAG mouse Rnf12 delta L502-L513
recombinant DNA reagent	FLAG-RNF12 ΔRING	MRC-PPU Reagents and Services	DU53417	pCAGGS puro 3XFLAG mouse Rnf12 M1-L543
recombinant DNA reagent	FLAG-RNF12 ΔBR	MRC-PPU Reagents and Services	DU53418	pCAGGS puro 3XFLAG mouse Rnf12 delta Y326-A423
recombinant DNA reagent	FLAG-RNF12 4xSA	MRC-PPU Reagents and Services	DU67399	pCAGGS puro 3XFLAG mouse Rnf12 S212/214/227/229A
recombinant DNA reagent	FLAG-REX1	MRC-PPU Reagents and Services	DU63525	pCAGGS puro 3XFLAG mouse Rex1
recombinant DNA reagent	REX1	MRC-PPU Reagents and Services	DU50852	pCAGGS puro mouse Rex1

recombinant DNA reagent	FLAG-USP26	MRC-PPU Reagents and Services	DU53288	pCAGGS puro 3FLAG mouse USP26
recombinant DNA reagent	FLAG-USP26	MRC-PPU Reagents and Services	DU67207	pCAGGS puro 3FLAG human USP26
recombinant DNA reagent	FLAG-USP26 V154I	MRC-PPU Reagents and Services	DU61166	pCAGGS puro 3FLAG human USP26 V154I
recombinant DNA reagent	FLAG-USP26 Q156H	MRC-PPU Reagents and Services	DU61169	pCAGGS puro 3FLAG human USP26 Q156H
recombinant DNA reagent	FLAG-USP26 L165S	MRC-PPU Reagents and Services	DU61170	pCAGGS puro 3FLAG human USP26 L165S
recombinant DNA reagent	FLAG-USP26 L364F	MRC-PPU Reagents and Services	DU61171	pCAGGS puro 3FLAG human USP26 L364F
recombinant DNA reagent	FLAG-USP29	MRC-PPU Reagents and Services	DU61201	pCAGGS puro 3FLAG human USP29
recombinant DNA reagent	FLAG-USP38	MRC-PPU Reagents and Services	DU49091	pCAGGS puro 3XFLAG mouse USP38
recombinant DNA reagent	FLAG-USP2	MRC-PPU Reagents and Services	DU61200	pCAGGS puro 3FLAG human USP2
recombinant DNA reagent	3FLAG hUSP29	MRC-PPU Reagents and Services	DU61201	pCAGGS puro 3FLAG USP29
recombinant DNA reagent	HA mouse RNF12 K9R K71R	MRC-PPU Reagents and Services	DU61792	pCAGGS puro HA mouse RNF12 K9R K71R
recombinant DNA reagent	HA mouse RNF12 K526R K544R K558R K561R	MRC-PPU Reagents and Services	DU61819	pCAGGS puro HA mouse RNF12 K526R K544R K558R K561R

transfected construct (*Mus Musculus*)	*Usp26* Δ/y CRISPR sense guide	MRC-PPU Reagents and Services	DU52887	pBABED P U6 mUSP26 ex9 KO sense
transfected construct (*Mus Musculus*)	*Usp26 Δ/y* CRISPR antisense guide	MRC-PPU Reagents and Services	DU52891	pX335 mUSP26 ex9 KO antisense
transfected construct (*Mus Musculus*)	HA-RNF12 WT-KI guide	MRC-PPU Reagents and Services	DU69536	CRISPR N-terminal HA-RNF12 KI guide to be used in conjunction with ssODN donor
biological sample (*Mus Musculus,* male)	Brain lysate	This paper		Adult mouse tissue lysate from the laboratory of Victoria Cowling, GRE division, Dundee
biological sample (*Mus Musculus,* male)	Heart lysate	This paper		Adult mouse tissue lysate from the laboratory of Victoria Cowling, GRE division, Dundee
biological sample (*Mus Musculus,* male)	Lungs lysate	This paper		Adult mouse tissue lysate from the laboratory of Victoria Cowling, GRE division, Dundee
biological sample (*Mus Musculus,* male)	Liver lysate	This paper		Adult mouse tissue lysate from the laboratory of Victoria Cowling, GRE division, Dundee
biological sample (*Mus Musculus,* male)	Kidney lysate	This paper		Adult mouse tissue lysate from the laboratory of Victoria Cowling, GRE division, Dundee
biological sample (*Mus Musculus,* male)	Spleen lysate	This paper		Adult mouse tissue lysate from the laboratory of Victoria Cowling, GRE division, Dundee
biological sample (*Mus Musculus,* male)	Testis lysate	This paper		Adult mouse tissue lysate from the laboratory of Victoria Cowling, GRE division, Dundee
biological sample (*Mus Musculus,* male)	Muscle lysate	This paper		Adult mouse tissue lysate from the laboratory of Victoria Cowling, GRE division, Dundee
biological sample (*Mus Musculus,* male)	Testis sections	This paper		Adult mouse tissue section from the laboratory of Ingolf Bach, UMMS, MA

antibody	Anti-RNF12 (Sheep polyclonal)	MRC-PPU Reagents and Services	Cat#S691D third bleed	(WB:1:1000)
antibody	Anti-RNF12 (Mouse monoclonal)	Novus Biologicals	Cat#H00051132-M01 RRID:AB_547742	(WB:1:1000 IF: 1:200)
antibody	Anti-REX1 (Rabbit polyclonal)	Abcam	Cat#ab28141 RRID:AB_882332	(WB: 1:1000)
antibody	Anti-USP26 (Sheep polyclonal)	MRC-PPU Reagents and Services	Cat#SA085 third bleed	(WB:1:1000 IF:1:1000 IHC: 1:100)
antibody	Anti-ERK1 (Mouse monoclonal)	BD Biosciences	Cat#610408 RRID:AB_397790	(WB: 1:1000)
antibody	Anti-ERK1 (Rabbit polyclonal)	Santa Cruz	Cat#SC-93 RRID:AB_631453	(WB: 1:1000)
antibody	Anti-ERK1 (Sheep polyclonal)	MRC-PPU Reagents and Services	Cat#S221B fourth bleed	(WB: 1:2000)
antibody	Anti-DAZL (Sheep polyclonal)	MRC-PPU Reagents and Services	Cat#S836B third bleed	(WB:1:1000 IF: 1:200)
antibody	Anti-DPPA3 (Rabbit polyclonal)	Abcam	Cat#ab19878 RRID:AB_2246120	(WB:1:1000 IF: 1:200)
antibody	Anti-Total Ubiquitin (Rabbit polyclonal)	DAKO	Cat#Z0458 RRID:AB_2315524	(WB: 1:1000)
antibody	Anti-K48 ubiquitin (Rabbit polyclonal)	Cell Signaling Technology	Cat#4289 RRID:AB_10557239	(WB: 1:1000)
antibody	Anti-HA-HRP (Rat monoclonal)	Roche	Cat#12013819001 RRID:AB_390917	(WB:1:5000)
antibody	Anti-FLAG-HRP (Mouse monoclonal)	Sigma Aldrich	Cat# A8592 RRID:AB_439702	(WB:1:5000)

antibody	Anti-Actin (Rabbit monoclonal)	Cell Signaling Technology	Cat#4970 (13E5) RRID:AB_2223172	(WB:1:1000)
antibody	Anti-HA (Rabbit polyclonal)	Abcam	Cat#ab9110 RRID:AB_307019	(IF: 1:1000)
antibody	Anti-FLAG (Mouse monoclonal)	Sigma Aldrich	Cat#F1804-50UG RRID:AB_262044	(IF: 1:500)
antibody	Anti-Mouse HRP (Horse polyclonal)	Cell Signaling Technology	Cat#7076S RRID:AB_330924	(WB: 1:10000)
antibody	Anti-Rabbit HRP (Goat polyclonal)	Cell Signaling Technology	Cat#7074S RRID:AB_2099233	(WB: 1:10000)
antibody	Anti-Sheep HRP (Donkey polyclonal)	Thermo Fisher	Cat#16041 RRID:AB_2534715	(WB: 1:10000)
antibody	Anti-Rabbit AlexaFluor 405 nm (Goat polyclonal)	Lifetech	Cat#A31556 RRID:AB_221605	(IF: 1:500)
antibody	Anti-Rabbit AlexaFluor 488 nm (Goat polyclonal)	Lifetech	Cat#A11008 RRID:AB_143165	(IF: 1:500)
antibody	Anti-Sheep AlexaFluor 488 nm (Donkey polyclonal)	Lifetech	Cat#A11015 RRID:AB_2534082	(IF: 1:500)
antibody	Anti-Mouse AlexaFluor 555 nm (Donkey polyclonal)	Lifetech	Cat#A31570 RRID:AB_2536180	(IF: 1:500)
antibody	Anti-REX1 (Sheep polyclonal)	MRC-PPU Reagents and Services	Cat# DA136 fourth bleed	(WB: 1:2000)
antibody	Anti-Phospho-Histone H3 (Ser 10) (Rabbit polyclonal)	Cell Signalling	Cat#9701	(WB: 1:1000)

antibody	Anti-Tubulin β 3 (TUBB3) (mouse monoclonal)	Biolegend	Cat#801213	(WB: 1:1000)
antibody	Anti-Ubiquitin K11 linkage (rabbit monoclonal)	Sigma Aldrich	Cat#MABS107-I	(WB: 1:1000)
antibody	Anti-Ubiquitin K63 (HWA4C4) (mouse monoclonal)	Sigma Aldrich	Cat#05-1313	(WB: 1:1000)
sequence-based reagent	*Xist*_F	This paper	PCR primers	GGATCCTGCTTGAACTACTGC
sequence-based reagent	*Xist*_R	This paper	PCR primers	CAGGCAATCCTTCTTCTTGAG
sequence-based reagent	*1700013H16 Rik*_F	This paper	PCR primers	GGAGTTGACATTAACCGTGCT
sequence-based reagent	*1700013H16 Rik*_R	This paper	PCR primers	CATTAAGCTGTGCCATTGCATC
sequence-based reagent	*Usp9y*_F	This paper	PCR primers	ATGGCAGGTTGCACATTCAC
sequence-based reagent	*Usp9y*_R	This paper	PCR primers	CAGTCCATCTTGATCATTTGG
sequence-based reagent	*Pramel3*_F	This paper	PCR primers	CCTTTTGCCTGTCTCCACATTGG
sequence-based reagent	*Pramel3*_R	This paper	PCR primers	CAGCCAGCATCCTGCCTTAAATC
sequence-based reagent	*Magea4*_F	This paper	PCR primers	GGCTCACCTATGATGGGATGCT
sequence-based reagent	*Magea4*_R	This paper	PCR primers	CTCACTGACACAGTTTCCTTGCG

sequence-based reagent	*Pramel7*_F	This paper	PCR primers	GTGAGGAATGAAGTATTGACCGT
sequence-based reagent	*Pramel7*_R	This paper	PCR primers	TCAGCCATGTGTCTACTCCATC
sequence-based reagent	*Gm773*_F	This paper	PCR primers	TCTGTTTCAGCAGTGGGATTTTGA
sequence-based reagent	*Gm773*_R	This paper	PCR primers	AGTGCTTTCAGGCTGTGGACCT
sequence-based reagent	*Usp26*_F	This paper	PCR primers	GCACTGGATGCTAAATGCAA
sequence-based reagent	*Usp26*_R	This paper	PCR primers	TGTGCTGAGTGCCTGTCCTA
sequence-based reagent	*Rhox13*_F	This paper	PCR primers	ACCGCCATTCCACTTCGCAC
sequence-based reagent	*Rhox13*_R	This paper	PCR primers	ATTGGGCACAGAGGTTGC
sequence-based reagent	*Rhox5*_F	This paper	PCR primers	ACTCGGAAGAACAGCATGATG
sequence-based reagent	*Rhox5*_R	This paper	PCR primers	CCCTGGTGCCACTATCCTT
sequence-based reagent	*Dazl*_F	This paper	PCR primers	TGGACCGAAGCATACAGACAGTGGT
sequence-based reagent	*Dazl*_R	This paper	PCR primers	TGATCAGATTTAAGCACTGCCCGAC
sequence-based reagent	*Dppa3*_F	This paper	PCR primers	GACCCAATGAAGGACCCTGAA
sequence-based reagent	*Dppa3*_R	This paper	PCR primers	GCTTGACACCGGGGTTTAG

sequence-based reagent	*Scml2*_F	This paper	PCR primers	ATCTTCCCAGTTGGATGGTG
sequence-based reagent	*Scml2*_R	This paper	PCR primers	CTGGGGCCTCTTCTTCATTT
sequence-based reagent	*Tdrd12*_F	This paper	PCR primers	GGGCTCTGATTAAGTCCATCATC
sequence-based reagent	*Tdrd12*_R	This paper	PCR primers	ACTTGGCAAAATCGACCAGGA
sequence-based reagent	*Usp29*_F	This paper	PCR primers	CACGTGCCTGACCCAGCTACTTG
sequence-based reagent	*Usp29*_R	This paper	PCR primers	AGCTATAGCGTTTCAGATGGA
sequence-based reagent	*Nanog*_F	This paper	PCR primers	CTCATCAATGCCTGCAGTTTTTCA
sequence-based reagent	*Nanog*_R	This paper	PCR primers	CTCCTCAGGGCCCTTGTCAGC
sequence-based reagent	*Klf4*_F	This paper	PCR primers	ACACTTGTGACTATGCAGGCTGTG
sequence-based reagent	*Klf4*_R	This paper	PCR primers	TCCCAGTCACAGTGGTAAGGTTTC
sequence-based reagent	*Fgf5*_F	This paper	PCR primers	GCTGTGTCTCAGGGGATTGT
sequence-based reagent	*Fgf5*_R	This paper	PCR primers	CACTCTCGGCCTGTCTTTTC
sequence-based reagent	*Gapdh*_F	This paper	PCR primers	CTCGTCCCGTAGACAAAA
sequence-based reagent	*Gapdh*_R	This paper	PCR primers	TGAATTTGCCGTGAGTGG

peptide, recombinant protein	Activin A	Peprotech	Cat#120-14P	
peptide, recombinant protein	bFGF	Peprotech	Cat#100-18B	
peptide, recombinant protein	BMP4	R&D Systems	Cat#314-BP-010	
peptide, recombinant protein	BMP8a	R&D Systems	Cat#1073-BP-010	
peptide, recombinant protein	EGF	R&D Systems	Cat#236-EG-200	
peptide, recombinant protein	SCF	R&D Systems	Cat#455-MC-010	
peptide, recombinant protein	LIF	MRC-PPU Reagents and Services	DU1715	GST-tagged LIF
peptide, recombinant protein	UBE1	MRC-PPU Reagents and Services	DU32888	
peptide, recombinant protein	UBE2D1 (UbcH5a)	MRC-PPU Reagents and Services	DU4315	
peptide, recombinant protein	FLAG-Ubiquitin	MRC-PPU Reagents and Services	DU46789	
peptide, recombinant protein	RNF12	MRC-PPU Reagents and Services	DU61098	
peptide, recombinant protein	USP2	MRC-PPU Reagents and Services	DU13025	
peptide, recombinant protein	GST	MRC-PPU Reagents and Services		

commercial assay or kit	EZNA microelute total RNA kit	Omega Biotek	Cat#R6834-02	
commercial assay or kit	DNAse Set RNAse free	Omega Biotek	Cat#E1091	
commercial assay or kit	iScript cDNA conversion kit	BioRad	Cat#170-8891	
chemical compound, drug	PD0325901	AXON	Cat#1408	Bain J. (2007) Biochem J. 408 297–315.
chemical compound, drug	CHIR99021	AXON	Cat#1386	Ring D.B. (2003) Diabetes 52 588
chemical compound, drug	MG132	SIGMA	Cat#M8699	Mroczkiewicz M, et al. Journal of Medicinal Chemistry 53(4), 1509-1518, (2010)
chemical compound, drug	Cycloheximide	SIGMA	Cat#C4859	Schneider-Poetsch, T. et al. (2010) Nat Chem Biol 6, 209-217.
software, algorithm	Image Studio	LICOR Biosciences		https://www.licor.com/bio/image-studio/
software, algorithm	Image Lab	Bio-Rad		https://www.bio-rad.com/en-uk/product/image-lab-software?ID=KRE6P5E8Z
software, algorithm	Image J	NIH		https://imagej.nih.gov/ij/download.html
software, algorithm	STAR software (v2.7.1a)	PMID: 23104886		https://github.com/alexdobin/STAR
software, algorithm	HTSeq (v0.11.2)	PMID: 25260700		https://htseq.readthedocs.io/en/master/
software, algorithm	SARTools (v1.6.9)	PMID: 27280887		https://github.com/PF2-pasteur-fr/SARTools

software, algorithm	DESeq2 (v1.24)	PMID: 25516281		https://bioconductor.org/packages/release/bioc/html/DESeq2.html
software, algorithm	GOstats (v2.50.0)	PMID: 17098774		https://www.bioconductor.org/packages/release/bioc/html/GOstats.html
software, algorithm	GraphPad Prism (v7.0c)	GraphPad Software Inc.		https://www.graphpad.com/scientific-software/prism/
software, algorithm	Proteome Discoverer v.2.0	Thermo Fisher		https://www.thermofisher.com/order/catalog/product/OPTON-30812?SID=srch-srp-OPTON-30812#/OPTON-30812?SID=srch-srp-OPTON-30812
software, algorithm	Mascot	Matrix Science		https://www.matrixscience.com/server.html
software, algorithm	Excel	Microsoft		https://www.microsoft.com/en-us/microsoft-365/excel

Data file S1. Gene Ontology associated with mRNAs increased by RNF12. This table is a complete list of Gene Ontology terms that are significantly enriched within the cohort of mRNAs that was significantly increased upon RNF12 reconstitution in *Rlim*$^{-/y}$ mESCs. This table is provided as an Excel file. Gene Ontology terms associated with gametogenesis or reproduction are highlighted in yellow.
